# Sleep loss induces cholesterol-associated myelin dysfunction

**DOI:** 10.1073/pnas.2523438123

**Published:** 2026-01-20

**Authors:** Reyila Simayi, Eleonora Ficiarà, Oluwatomisin Faniyan, Antonio Cerdán Cerdá, Amina Aboufares El Alaoui, Rosamaria Fiorini, Adele Cutignano, Fabiana Piscitelli, Aroa S. Maroto, Alexandra Santos, Federico Del Gallo, Luisa de Vivo, Silvia De Santis, Michele Bellesi

**Affiliations:** ^a^School of Pharmacy, University of Camerino, Camerino 62032, Italy; ^b^Center for Neuroscience, University of Camerino, Camerino 62032, Italy; ^c^School of Biosciences and Veterinary Medicine, University of Camerino, Camerino 62032, Italy; ^d^Instituto de Neurociencias (Consejo Superior de Investigaciones Científicas - Universidad Miguel Hernández), San Juan de Alicante 03550, Spain; ^e^Department of Life and Environmental Sciences, Marche Polytechnic University, Ancona 60100, Italy; ^f^Istituto di Chimica Biomolecolare–Consiglio Nazionale delle Ricerche, Pozzuoli 80078, Italy; ^g^School of Physiology, Pharmacology and Neuroscience, University of Bristol, Bristol BS8 1TD, United Kingdom

**Keywords:** sleep, myelin, oligodendrocyte, white matter

## Abstract

The increasing prevalence of sleep deprivation poses a public health challenge in modern society. Manifestations of reduced alertness, such as slowed reaction times and increased errors, are well-documented behavioral indicators of sleep loss (SL). Yet, the biological consequences of sleep deprivation and their role in behavioral impairment remain elusive. Our study reveals significant effects of sleep deprivation on myelin integrity. As a result, we identify increased conduction delays in nerve signal propagation, hindered interhemispheric synchronization, and impaired cognitive and motor performance associated with SL. By profiling oligodendrocyte transcriptome and lipidome, we observe SL-induced endoplasmic reticulum stress and lipid metabolism dysregulation, particularly affecting cholesterol homeostasis. Boosting cholesterol transport to myelin sheaths prevents SL effects on nerve signal propagation and behavior. Our findings highlight a possible role of oligodendrocyte cholesterol dysregulation in behavioral deficits associated with SL and unveil a novel target for intervention.

## Significance

Although the behavioral consequences of sleep loss (SL) are well known, the underlying biology has remained elusive. This study identifies oligodendrocytes as key mediators by linking sleep deprivation to impaired myelin integrity, slowed nerve conduction, and behavioral deficits. Importantly, cholesterol imbalance in oligodendrocytes emerges as a novel mechanistic pathway through which SL impairs myelin function and neural signal propagation, ultimately driving cognitive and behavioral impairments.

While the functions of sleep remain to be fully understood, it is clear that even a single missed night of sleep or consistently shortened sleep durations over extended periods can importantly affect brain functioning and behavior. Slow reaction times and increased errors of omission at the psychomotor vigilance task (PVT) are the most objective changes in alertness associated with sleep loss (SL) (1, 2). These changes can coincide with sleep episodes intruding into wakefulness and a slowdown of waking electroencephalographic (EEG) rhythms (3).

Considerable work has been undertaken to determine the characteristics of the “neural fatigue” and to uncover the mechanisms causing neural network anomalies that result in compromised behavior. For instance, it has been found that SL can induce brief periods of neuronal silence (i.e., local off periods) in an otherwise awake rat brain. This cellular effect has been related to impaired motor performance in a food-pellet reaching task during sleep deprivation (4). Similarly, in humans who had intracranial electrodes implanted to monitor individual neurons, there were noticeable local changes in neuronal activity just before attention lapses occurred during the PVT (5).

While most of the attention has been focused on neurons, other brain cells have received little consideration, despite the evidence showing that glial cells can respond to SL with molecular changes and extensive structural modifications (6, 7). For instance, electron microscopy investigations in mice have shown that chronic sleep restriction can lead to decreased myelin thickness and changes in the nodal region’s geometry of callosal axons, implicating adaptive remodeling of oligodendrocytes (8, 9).

Emerging evidence has established the remarkable adaptability of oligodendrocytes in modulating myelin structure and function (10). These cells dynamically adjust the thickness and extent of myelin sheaths in response to neural activity (11), environmental changes (12), and learning (13). These changes can, in turn, fine-tune the speed at which action potentials travel along axons, optimizing brain rhythms and connectivity within and across neural circuits and leading to improved cognitive functions and behavioral performance (14, 15). Here, we explore the effects of SL on white matter (WM) and oligodendrocyte function, investigating its impact on neuronal signal conduction, synchronization, and its contribution to the behavioral deficits associated with sleep deprivation. Moreover, we identify altered cholesterol metabolism in oligodendrocytes as one of the potential biological mechanisms responsible for the impaired behavioral performance observed after sleep deprivation.

## Results

### SL Impacts Myelin Integrity.

**Fig. 1.**
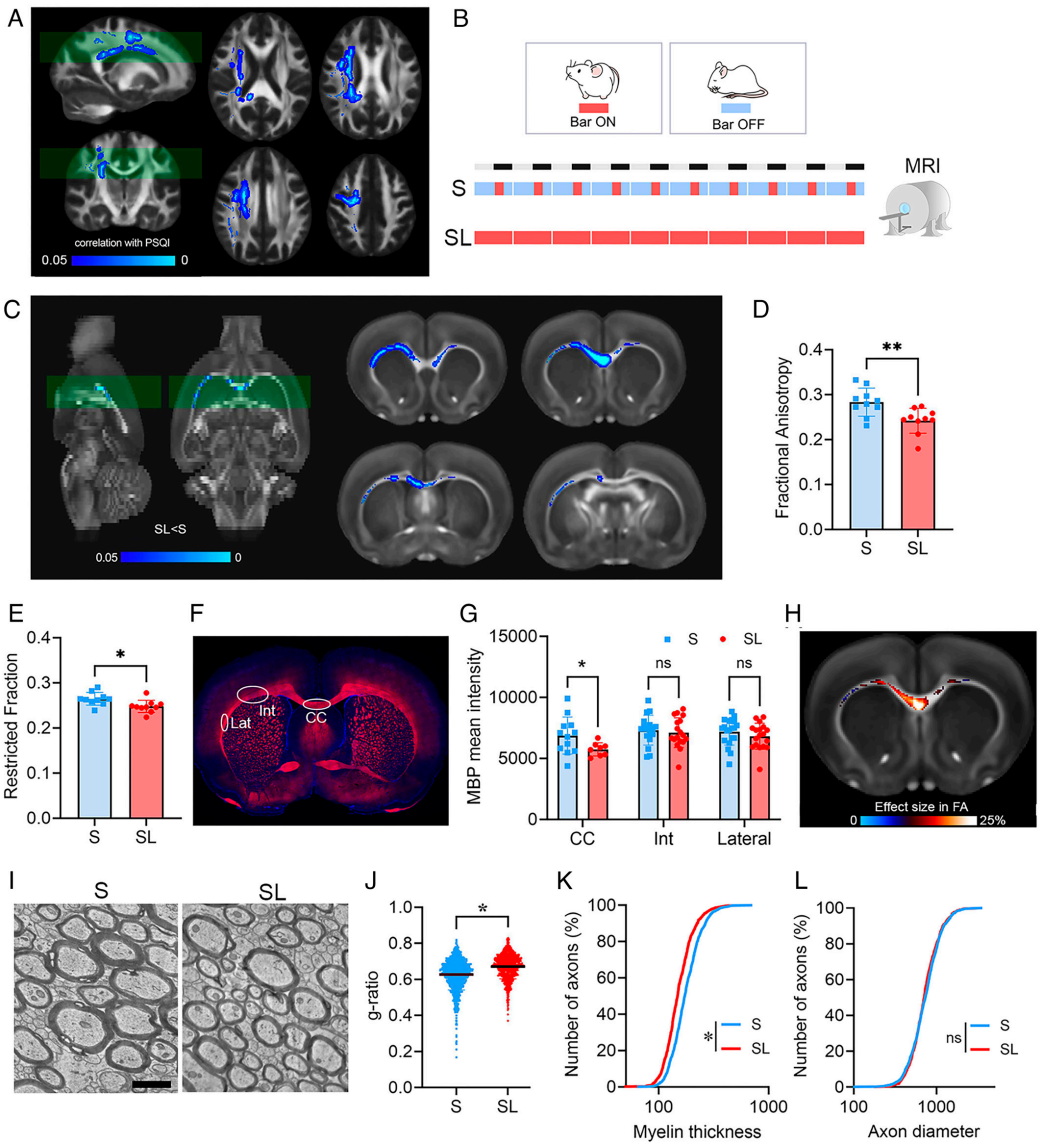
Widespread and inter-species effects of SL on brain WM. (*A*) Significant association between FA and PSQI in the Human Connectome Project (HCP) database (n = 185) (20), superimposed on the FA template. Blue-light blue: significant negative association (*P* < 0.05). The opposite contrast is not significant. (*B*) Rat experimental design [sleep (S), n = 10; SL, n = 10]. “Bar ON” marks the active phase of the sleep restriction apparatus; “Bar OFF” indicates periods when the bar was inactive, allowing sleep. (*C*) Significant differences of FA in SL relative to S, superimposed on the FA template. Blue-light blue: significant reduction in SL (*P* < 0.05). The opposite contrast is not significant. (*D* and *E*) Quantification of FA (*D*) and FR (*E*) in S (blue) and SL (red) rats. *P < 0.05; **P < 0.01. (*F*) Example of MBP staining (red) and DAPI (blue) in a S rat frontal section. White circles indicate regions of MBP quantification. (Scale bar, 2 mm.) (*G*) Quantification of MBP staining in corpus callosum (CC), intermediate (Int), and lateral (Lat) subcortical WM. *P < 0.05. ns: not significant. (*H*) Voxel-wise FA effect size computed as the percentage change in average FA between the SL and S groups, normalized to the average FA of the S group. (*I*) Representative electron microscopic pictures illustrating myelinated axons from CC of a S and a SL rat. (Scale bar, 1 µm.) (*J*) g-ratio quantification for myelinated fibers of S (n = 1,132, three rats) and SL (n = 900, three rats) rats. *P < 0.05. (*K* and *L*) Cumulative frequency distributions for myelin thickness (*K*) and axon diameter (*L*) data. *P < 0.05. ns: not significant.

Poor sleep quality and short sleep duration have been associated with MRI alterations in WM microstructure in multiple brain regions (16–18). In the first analysis, we sought to confirm this association in a large sample of healthy individuals (n = 185) by quantifying the relation between WM microstructural integrity and the Pittsburgh Sleep Quality Index (PSQI), a well-recognized metric of sleep quality. We found a significant negative correlation between the PSQI and MRI makers of WM integrity indicating that poor sleep quality was related to lower microstructural integrity. Such effect was more pronounced and widespread in the WM skeleton when microstructural integrity was assessed using the fractional anisotropy (FA, Fig. 1*A*). Other structural markers such as the restricted function (FR) derived from the CHARMED model (19) also showed a significant but weaker association with PSQI (*SI Appendix*, Figs. S1 and S2).

To confirm the causal link between WM microstructural integrity and sleep disruption, in a second experiment we measured WM microstructural integrity in rats that had been subjected to sleep restriction (SL group) and in matched normal sleeping controls (S) using structural MRI. We first validated with polysomnography an automatic sleep restriction approach (*SI Appendix*, Fig. S3) and then we used it to sleep restrict rats for 10 d prior the MRI scanning (Fig. 1*B*). Voxel wise analysis of the WM revealed a widespread decrease in FA (*P* = 0.006) and FR (*P* = 0.015) in SL animals relative to controls, which was indicative of broad and bilateral reduced microstructural integrity due to SL (Fig. 1 *C*–*E*).

Since MRI structural markers lack the specificity to determine whether reduced structural integrity results from myelin loss or axonal damage, we performed immunostaining for myelin basic protein (MBP), a marker highly expressed in myelin sheaths, on brains following MRI scans (Fig. 1*F*). Quantification of MBP immunoreactivity in regions showing decreased FA revealed a significant reduction in MBP levels in SL rats compared to *S* controls in the corpus callosum (CC) (*P* = 0.045). However, MBP expression in the lateral subcortical WM was not significantly affected [Intermediate (Int): *P* = 0.85; Lat: *P* = 0.55; Fig. 1*G*], consistent with the smaller FA effect sizes observed in these regions at the MRI scan (Fig. 1*H*).

At the ultrastructural level, myelinated axons of CC of SL rats showed an increased g-ratio relative to controls (*P* = 0.02; Fig. 1 *I* and *J*). This change was largely driven by overall reduced myelin thickness rather than variations in axonal diameter (Fig. 1 *K* and *L*). Furthermore, we found a trend toward a higher proportion of unmyelinated axons in SL rats compared to S rats (unmyelinated/total axons, *P* = 0.1; *SI Appendix*, Fig. S4*A*).

Finally, we evaluated the impact of SL on oligodendrocyte precursor cell (OPC), pre-myelinating (preOL), and mature oligodendrocyte (OL) densities in the subcortical WM and CC. Results showed a significant reduction in OPC density (*P* = 0.0002), consistent with previous studies showing a decrease in OPC proliferation following sleep deprivation (6). Densities of preOL showed a trend towards an increase in SL relative to S (*P* = 0.051), whereas OL densities remained mostly unaffected by SL (*P* = 0.32; *SI Appendix*, Fig. S4 *B* and *C*).

These findings collectively demonstrated that SL induced widespread reductions in myelin integrity, marked by thinner myelin sheaths without major modifications of the axon size. This effect was also associated with reduced OPCs density.

### SL Increases Conduction Delays.

**Fig. 2.**
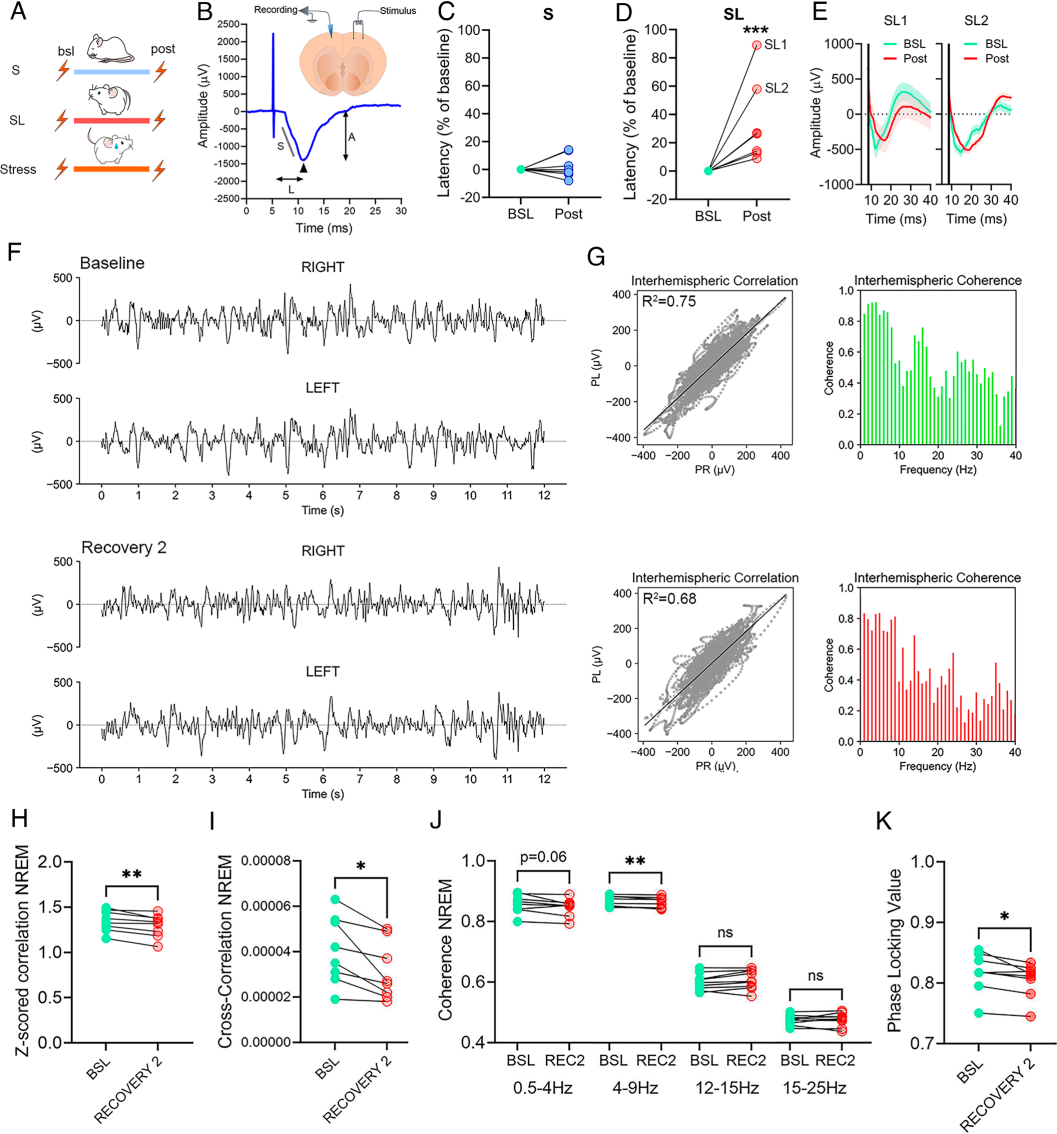
SL increases conduction delays and reduces interhemispheric synchronization. (*A*) Experimental design. (*B*) Example of an evoked cortico-cortical transcallosal response in a S rat. L: peak latency; A: peak amplitude; S: slope. Arrows indcates the negative peak of the early component of the evoked response. The picture above indicates the location of the stimulation and recording LFP electrodes in the frontal cortex. (*C* and *D*) Mean latency values of the early component negative peak for S (*C*, blue, n = 8) and SL (*D*, red, n = 8) individual rats. Values are represented as % relative to their own baseline. ***P < 0.001. SL1 and SL2 indicate the two rats showing the largest latency changes. (*E*) Raw stimulation traces from SL1 and SL2 recorded before (BSL, green) and after (Post, red) SL. The black vertical line indicates stimulus onset. Thick lines represent the mean across trials, and shaded areas indicate the SEM. (*F*) Two examples of raw 12-s EEG records of NREM sleep from the right and left parietal derivations for a SL rat during baseline (*Upper*) and during the second day of recovery after sleep restriction (*Lower*). (*G*) *Left*: voltage values of the left derivation were plotted as a function of the right derivation for corresponding consecutive sampling intervals for the 12-s records depicted in (*F*). The bold line represents a linear regression. *Right*: interhemispheric coherence spectra for frequencies between 0.5 and 40 Hz of the corresponding 12-s records depicted in (*F*). (*H* and *I*) Interhemispheric *z*-scored correlation (*H*) and cross-correlation (*I*) values for SL rats (n = 8) computed for baseline and the second day of recovery after sleep restriction (recovery 2). *P < 0.05. **P < 0.01. (*J* and *K*) Interhemispheric coherence (*J*) and phase-locking (*K*) values computed for baseline and recovery 2 (REC2). *P < 0.05. **P < 0.01.

While both ultrastructural and MRI findings demonstrated a reduction in myelin content after sleep deprivation, whether myelin function is also altered in vivo has never been tested. Based on MRI and ultrastructural studies, we postulated that SL-induced myelin modifications could result in delayed signal transmission, i.e., reduced speed of action potentials travelling along the axons. Therefore, we measured in vivo the latency of transcallosal cortical local field potentials (LFPs) evoked by the electrical stimulation of the contralateral homotopic cortex, which is inversely related to the signal conduction velocity along the connections. To evaluate changes in response latency as a function of sleep and SL, we implanted two groups of rats with bipolar concentric electrodes for electrical stimulation and chronic intracortical LFP recordings (Fig. 2*A*). We recorded LFPs from the frontal cortex after electrical stimulation of the contralateral frontal cortex before (baseline) and after sleep manipulation (post session, Fig. 2*B*). We then measured the latency of the first negative component of the transcallosal evoked response (Fig. 2*B*). By comparing the post session with the baseline, we found that the latency of the early negative component was delayed by 32.8% ± 27.3% (*P* < 0.0001) in the SL group, while it did not change in S rats (*P* = 0.99), thus confirming SL-dependent increase in conduction delay in vivo (Fig. 2 *C*–*E*). Increased conduction delays could also modify the amplitude and the slope of the evoked potential, especially if the decrease in myelination is heterogeneous among callosal bundles. However, despite the large variability among the animals, on average neither the amplitude nor the slope of the evoked response significantly changed after SL (Amplitude: *P* = 0.81, Slope: *P* = 0.96; *SI Appendix*, Fig. S5).

While we intentionally used automated sleep restriction to reduce hands-on interaction with the animals, sleep deprivation is broadly recognized as a stressful condition, often elevating stress markers like corticosterone levels (8). Additionally, there is evidence suggesting that stress can negatively affect myelin (21). To understand whether the observed functional effects on myelin were related to the stress from the sleep restriction procedure, we evaluated cortico-cortical evoked responses in a separate rat group before and after subjecting them to 10 d of mild chronic stress. This approach, which involved systematic exposure to different mild stressors, is a standard method for assessing the impact of chronic stress. The analysis of the evoked responses did not reveal any significant difference in latency, amplitude, or slope between baseline and post stress sessions (Latency: *P* = 0.56; Amplitude: *P* = 0.5; Slope: *P* = 0.99; *SI Appendix*, Fig. S6 *A*–*C*). In addition, plasma corticosterone levels were comparable across experimental conditions (*P* = 0.26; *SI Appendix*, Fig. S6*D*), consistent with previous evidence that chronic stress, particularly when repetitive or occurring at fixed times, elicits blunted corticosterone responses (22, 23).

Taken together, these findings demonstrate that SL robustly increases transcallosal conduction delays in vivo, and that this effect is unlikely to be explained by the mild chronic stress tested here.

### SL Affects Interhemispheric Neural Synchronization.

We showed that SL can result in a reduced nerve pulse propagation speed compared to normal sleeping animals. Therefore, we posited that this could cause broad delays in the timing of action potentials (15), especially for those travelling on the heavily myelinated fibers of the CC. As a result, interhemispheric synchronization of neuronal activity would be reduced in SL rats. To test this hypothesis and minimize the number of implanted rats, we used the electroencephalographic (EEG)-implanted rats that were intended for testing the sleep restriction apparatus (*SI Appendix*, Fig. S3). We first measured the correlation and coherence of the EEG signals between the two hemispheres during 24 h of baseline and after 4 d of sleep restriction (Fig. 2 *F* and *G* and *SI Appendix*, Fig. S3*A*). We found that the temporal correlation was reduced during NREM sleep, while it was unchanged during wake and REM sleep (NREM: Pearson’s correlation: *P* = 0.004; cross-correlation: *P* = 0.011. Wake: Pearson’s correlation: *P* = 0.75; cross-correlation: *P* = 0.67. REM: Pearson’s correlation: *P* = 0.4; cross-correlation: *P* = 0.99; Fig. 2 *H* and *I* and *SI Appendix*, Fig. S7 *A–D*). Further analysis of coherence confirmed that the main effects occurred during NREM sleep, showing a general decrease in coherence within the delta and theta frequency ranges (*P* = 0.055 and *P* = 0.01, respectively), with no significant changes in higher frequencies (*P* = 0.2 for sigma; *P* = 0.74 for beta; Fig. 2*J*). Consistently, during NREM sleep we also observed a reduction in phase-locking value (PLV) within the delta-theta range (*P* = 0.02; Fig. 2*K*).

In wakefulness, coherence analysis revealed a significant reduction in beta-band coherence after SL (*P* = 0.01), while other frequency bands remained unchanged (Delta: *P* = 0.25; Theta: *P* = 0.99; Sigma: *P* = 0.2; *SI Appendix*, Fig. S7*E*). No significant differences were observed during REM sleep (Delta: *P* = 0.46; Theta: *P* = 0.15; Sigma: *P* = 0.46; Beta: *P* = 0.25; *SI Appendix*, Fig. S7*F*).

Thus, SL impaired interhemispheric synchronization of neuronal activity.

### SL Alters ER and Lipid Homeostasis.

**Fig. 3.**
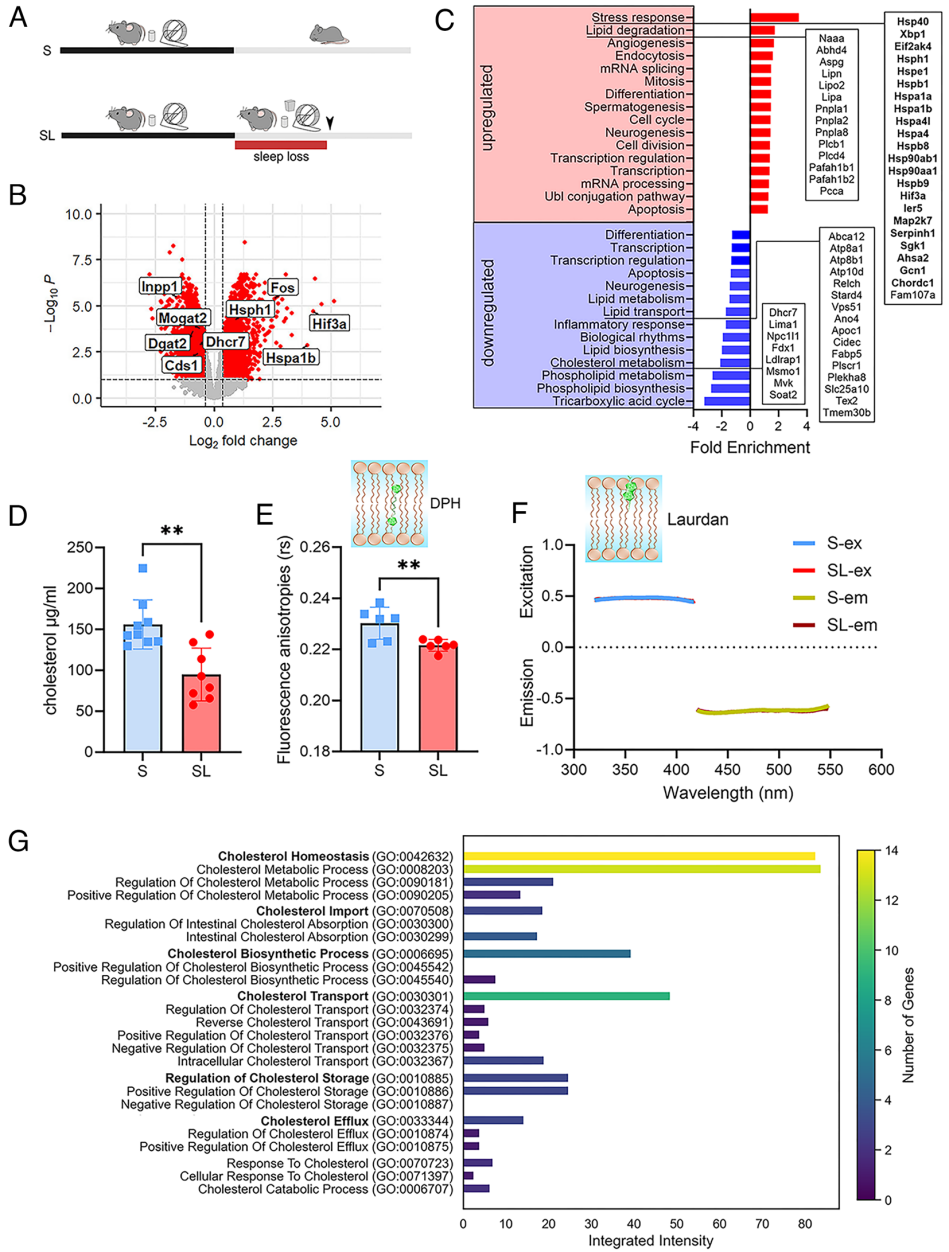
SL induces transcriptional changes in oligodendrocytes and alters cholesterol homeostasis affecting myelin membranes fluidity. (*A*) Experimental design of transcriptomics and lipidomic studies. (*B*) Volcano plot of the significant up-regulated and down-regulated transcripts (red) because of SL (S, n = 6; SL, n = 6). Some transcripts related to ER stress and lipid homeostasis are highlighted in bold. (*C*) Functional enrichment analysis identifying significant and highly enriched categories of up-regulated (red) and down-regulated (blue) genes. Categories of up (red) and down-regulated (blue) genes are ranked according to their enrichment score. Genes belonging to some key categories are highlighted in the frames. Note that in the stress response category genes related to ER stress are in bold. (*D*) Cholesterol levels as measured in the purified myelin fractions of S (n = 10) and SL (n = 8) animals with LC–MS. **P < 0.01. (*E*) Fluorescence anisotropies (rs) inversely related to core membrane fluidity in the purified myelin fractions of S (n = 6) and SL (n = 6) animals. **P < 0.01. (*F*) Laurdan excitation and emission spectra measurements in the purified myelin fractions of S and SL animals indicating fluidity stability in the outer part of the myelin membranes. For (*E*) and (*F*), the small *Inset* indicates the location in the plasma membrane where the fluorescent probe binds. (*G*) Cholesterol-related functional annotation analysis for down-regulated genes. In bold are the “parent” GO terms. Integrated intensity was computed as the sum of the average expression of the genes involved in that specific category.

Morphological and functional data suggest that SL can lead to myelin deficits, which may be attributed to an overall dysfunction of the oligodendroglia. For a more in-depth understanding, we interrogated a publicly available gene dataset that was previously obtained in 2’,3’-cyclicnucleotide3’-phosphodiesterase(CNP)-eGFP-L10a mice using TRAP technology combined with microarray analysis to tag polysomes and immunoaffinity purify oligodendrocyte specific mRNAs (6). By comparing data from sleeping mice (S, 6 to 7 h of sleep during the light phase; n = 6) with data obtained from sleep deprived mice (4 h of SL during the light phase through exposure to novel objects, n = 6; Fig. 3*A*), we identified 5,035 probe sets differentially expressed because of SL [8.9% of 45,101 probe sets; false discovery rate (FDR) = 1%], representing 3,448 unique genes, including 1,889 upregulated and 1,559 downregulated genes (Fig. 3*B* and Dataset S1). Upregulated and downregulated genes were clustered to distinct biological processes using the gene annotation enrichment analysis (Fig. 3*C*). Among the upregulated genes, an importantly enriched class was related to endoplasmic reticulum stress. Specifically, we found genes associated with PERK and IRE1 arms of the unfolding protein response (UPR; e.g. Xbp1, Hif3a, Eif2ak4) and to the ER associated degradation (ERAD) such as Hspa1a, Hsp90aa1, and Hsph1. Other upregulated genes were involved in lipid degradation of plasma membrane phospholipids (e.g. Plcb1 and Plcd4) and lysophospolipids (e.g., Naaa, Abhd4, Aspg, Lpcat2, Lipa). Of note, Lipa codes for a cholesterol ester hydrolase and is implicated in the hydrolysis of cholesteryl esters and triglycerides (TG) (24). The most enriched classes of downregulated genes were related to lipid biosynthesis and trafficking, which included genes involved in the synthesis of cholesterol (e.g., Dhcr7, Msmo1, Mvk) and glycerophospholipids (PC-PE-PI) (e.g., Digat2, Elovl7). The class of lipid transport included genes participating in the processes of phospholipid scrambling from one side of the plasma membrane to the other one (e.g. Atp8b1, Plscr1, Tmem30b) and other genes implicated in intracellular cholesterol trafficking (Abca12, Relch, Stard4, Apoc1) and ceramide transport (e.g., Plekha8 and Tex2). Notably, downregulation of Abca12 limits cholesterol efflux, while reduced expression of Relch and Stard4 are found to decrease non-vesicular cholesterol transport (25, 26). In summary, the analysis of gene expression in oligodendrocytes revealed that SL significantly impaired ER and lipid homeostasis, particularly affecting cholesterol metabolism.

### SL Reduces Myelin Cholesterol.

Guided by the transcriptomic analysis, we employed liquid chromatography–mass spectrometry (LC–MS) to quantify cholesterol levels in highly purified myelin fractions from mice subjected to 6 h of SL, induced by novel object exposure during the light cycle, and control mice allowed to sleep ad libitum during the same period (S). This analysis demonstrated a marked reduction in myelin cholesterol concentration in SL mice compared to S mice (*P* = 0.0011, Fig. 3*D*).

Optimal membrane fluidity and curvature in myelin necessitate high cholesterol levels. This ensures membrane stability, minimizes ion leakage, and reinforces the insulating properties of myelin membranes (27). To verify whether the drop in cholesterol correlated with changes in myelin membrane fluidity, we performed spectrofluorimetric assessments using 1,6-diphenyl-1,3,5-hexatriene (DPH), integrated into the hydrophobic lipid section, and 2-dimethylamino-(lauroyl)-naphtalene (Laurdan), positioned at the juncture between the hydrophobic and hydrophilic areas of the membrane. Examination of DPH’s steady-state fluorescence anisotropies (rs) showed a notable reduction in myelin derived from the brains of SL mice, pointing to enhanced fluidity in the inner segment of the bilayer (*P* = 0.0097; Fig. 3*E*). However, Laurdan’s quantitative analysis did not exhibit any distinctions between S and SL in both excitation and emission GP spectra (Excitation: *P* = 0.98; Emission: *P* = 0.71; Fig. 3*F*), implying that the fluidity of the external segment of the membrane was not affected by the cholesterol reduction.

To further explore SL-dependent molecular mechanisms affecting cholesterol homeostasis in oligodendrocytes, we performed a targeted transcriptomic analysis focused on brain-specific cholesterol-related pathways. Using the GO biological process annotations filtered by the term “cholesterol,” we conducted differential expression analysis on this selective dataset using a FDR of 5%. As illustrated in Fig. 3*G*, we found that the most notable differences belonged to the GO terms related to cholesterol homeostasis and transport, while biosynthesis and storage were less represented, suggesting a potential impairment of cholesterol intracellular transport and, to a lesser extent, cholesterol production.

Moreover, to assess whether the observed changes in cholesterol-related transcripts were specific to oligodendrocytes, we compared the magnitude of fold changes for primary cholesterol-related genes across three datasets: oligodendrocytes, all other brain cell types excluding oligodendrocytes (unbound fraction; see *SI Appendix*), and astrocytes (7). This comparison was made possible by leveraging datasets generated using the same BACTRAP methodology, allowing for a direct and reliable assessment of cell type–specific transcriptional responses. We found that many transcripts representing key cholesterol pathways were altered in the oligodendrocyte samples, but showed minimal changes in other cell types (*SI Appendix*, Fig. S8).

Finally, since gene enrichment analysis revealed significant lipid modifications beyond altered cholesterol metabolism, we performed untargeted lipidomics to comprehensively characterize the lipid composition of S and SL myelin-enriched fractions (Dataset S2). Differential expression analysis highlighted several lipids that changed their concentration because of SL, including acylcarnitine (AcCa), lysophosphatidylcholine (LPC), and glycerophospholipids (phosphatidylcholine [PC], phosphatidylethanolamine [PE], phosphatidylinositol [PI]) among the upregulated, and triglycerides (TG) and sulfatides (ST) among the downregulated (*SI Appendix*, Fig. S9*A*). However, none of them remained significant after multiple comparisons’ correction (*SI Appendix*, Fig. S9*B*), with the only exception of LPC (18:3), which showed a trend towards an increase in SL (*P* = 0.066).

In summary, these findings indicated SL markedly reduces cholesterol levels in myelin-enriched preparations, resulting in increased myelin membrane fluidity. The observed reduction in myelin cholesterol was related to deficits in intracellular trafficking and transport mechanisms. Moreover, the impact of SL on major cholesterol-related transcripts was more pronounced in oligodendrocytes than in other brain cell types. Finally, SL was associated with minimal changes of other lipid species.

### Cyclodextrin Prevents SL Deficits.

**Fig. 4.**
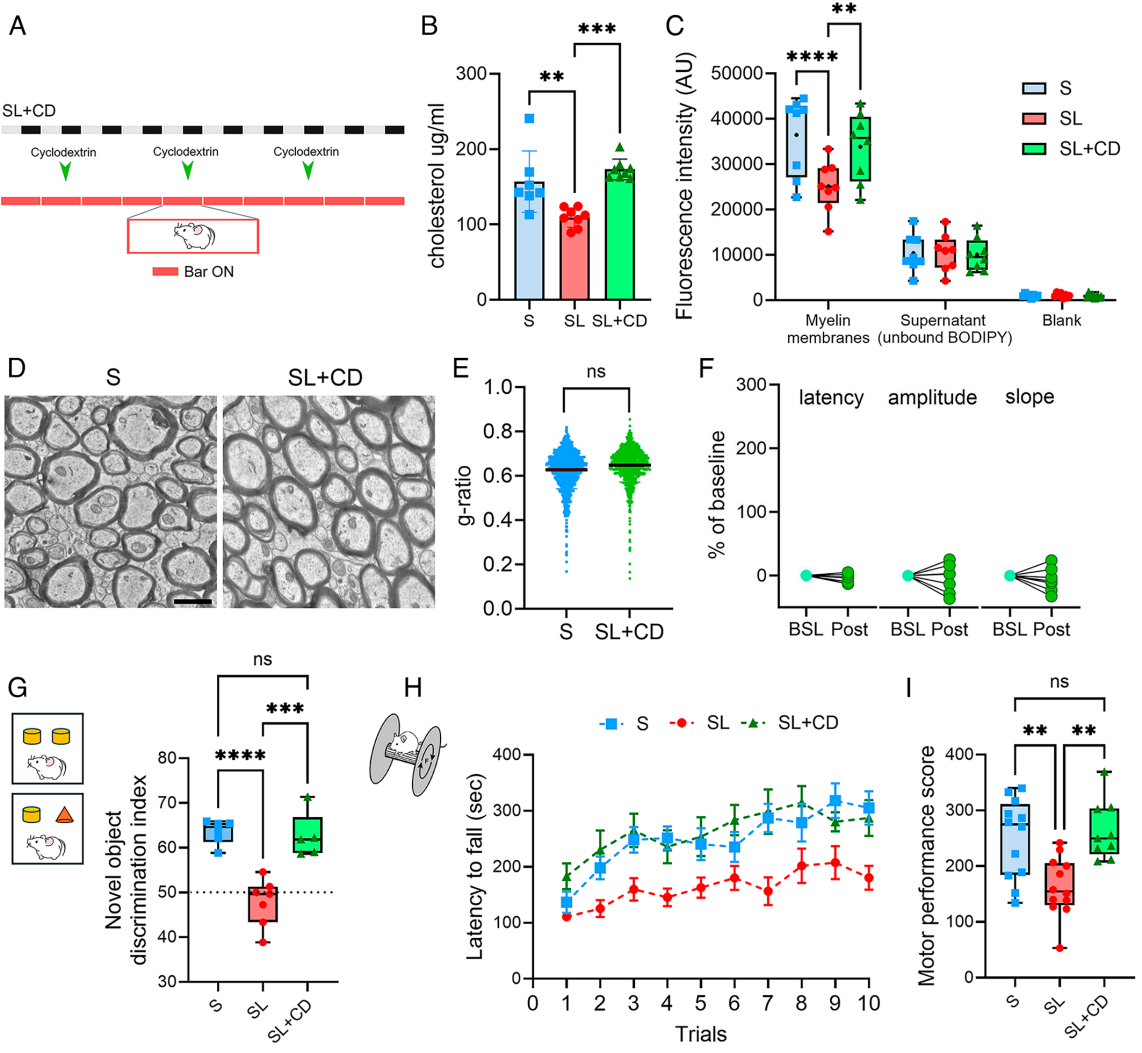
SL-related conduction delays and behavior modifications are prevented by aiding cholesterol transport. (*A*) Experimental design. (*B* and *C*). Cholesterol levels as measured in the purified myelin fractions S (n = 8), SL (n = 8), and SL + CD (n = 8) animals with LC–MS (*B*) and with BODIPY-cholesterol assay (*C*). (*D*) Representative electron microscopic pictures illustrating myelinated axons from CC of a S and a SL + CD rat. (Scale bar, 1 µm.) (*E*) g-ratio quantification for myelinated fibers of S (n = 1,132, three rats) and SL + CD (n = 1,126, three rats). *P < 0.05. (*F*) Mean latency, amplitude, slope values of the early component negative peak for SL individual rats that were treated with cyclodextrin (n = 7). Values are represented as % relative to their own baseline. (*G*) Scheme of the novel-object recognition task with S (n = 6), SL (n = 7), and SL + CD (n = 5) rats. Novel discrimination index was calculated by dividing the time that the animal explored the new object by the total time that the animal interacted with either object. The dashed line represents equal time spent exploring both the novel and familiar objects. Data points represent individual rats. Lines within the box blot represent mean values. ***P < 0.001. ****P < 0.0001. ns: not significant. (*H*) Scheme of the rotarod task with S (n = 12), SL (n = 12), and SL + CD (n = 9) rats. Latency to fall (in s) during 10 trials of the accelerating rotarod experiment. (*I*) Motor performance score of S, SL, and SL+CD rats. **P < 0.01. ns: not significant.

In mouse LC–MS analyses, SL was found to reduce cholesterol levels in myelin, while transcriptomic studies showed that cholesterol homeostasis and transport were the primary cellular pathways affected by SL in oligodendrocytes. We reasoned that by boosting cholesterol redistribution during SL we could minimize or prevent myelin dysfunctions and restore optimal conduction velocity in rats (Fig. 4*A*).

To test this, we first measured cholesterol levels in myelin-enriched fractions obtained from SL and S rats to confirm that SL could affect myelin cholesterol levels not only in mice but also in rats. We used a combination of methods based on LC–MS and BODIPY–cholesterol assay. Both approaches revealed a significant reduction of myelin cholesterol in SL preparations relative to S (LC–MS: *P* = 0.0039; BODIPY–cholesterol assay: *P* < 0.0001; Fig. 4 *B* and *C*). In the LC–MS analysis, this decrease remained consistent across alternative normalization methods (*SI Appendix*, Fig. S10). Notably, the reduction in cholesterol was specific to oligodendrocytes, as BODIPY–cholesterol fluorescence levels measured in astrocytes and neurons were not affected by SL (*SI Appendix*, Fig. S11).

Then, we administered 2-hydroxypropyl-β-cyclodextrin (cyclodextrin) to a group of rats and measured its effects on cholesterol levels, myelin ultrastructure, in vivo conduction delay before and after SL. Finally, we evaluated the impact of SL and cyclodextrin treatment on behavior. Cyclodextrin has demonstrated ability to bind and solubilize cholesterol, facilitating its redistribution from lysosomal storage organelles to other cellular compartments such as the plasma membrane (28, 29, 30). Importantly, recent studies demonstrated that cyclodextrin can aid cholesterol transport to myelin thus improving myelination in oligodendroglia (31). Building on this evidence, three subcutaneous injections of cyclodextrin were delivered over 10 d of sleep restriction. This treatment was able to prevent SL induced changes in cholesterol levels (*P* = 0.43; Fig. 4 *A*–*C*) and in the ultrastructure of myelin (g-ratio, *P* = 0.17; Fig. 4 *D* and *E*). Interestingly, cyclodextrin did not reduce the percentage of unmyelinated axons (unmyelinated/total axons, *P* = 0.016) or fully restore OPC density in the CC (*P* = 0.012; *SI Appendix*, Fig. S12). However, cyclodextrin prevented the SL effect on conduction delay (*P* = 0.83), with no effect on amplitude and slope of the evoked responses (Amplitude: *P* = 0.9, Slope: *P* = 0.91; Fig. 4*F*). Furthermore, cyclodextrin averted the deterioration of cognitive functions and motor behavior induced by SL. Sleep-restricted rats showed a marked reduction in the novel object discrimination index (*P* < 0.0001; Fig. 4*G*) and diminished motor performance relative to normally sleeping rats (*P* = 0.0037; Fig. 4 *H* and *I*). In contrast, cyclodextrin-treated rats performed comparably to controls (NOR: *P* = 0.98; rotarod: *P* = 0.87) and significantly better than untreated SL animals (NOR: *P* = 0.0002; rotarod: *P* = 0.002; Fig. 4 *G*–*I*).

These results suggest that aiding cholesterol transport restored normal myelin functions and prevented behavioral deficits in SL rats.

## Discussion

We observed a reduction of WM integrity in individuals with poor sleep quality and in rats subjected to sleep restriction. In rats, this reduction in myelin integrity was linked to structural remodeling of myelin thickness, confirming and expanding earlier observations of myelin ultrastructural changes documented in sleep restricted mice (8).

Oligodendrocyte vulnerability to SL may be linked to ER stress, as indicated by the significant upregulation of many ER stress-related transcripts following SL. It is known that sleep deprivation can intensify the demand for cellular resources, amplify metabolism, thereby producing significant oxidative stress, or even result in ER calcium depletion. All these mechanisms finally lead to ER stress (32, 33). Given the ER central role in lipid biosynthesis and trafficking, its dysfunction can disrupt lipid homeostasis within the cell (31, 34, 35, 36). In oligodendrocytes, lipid homeostasis is finely tuned and is essential for maintaining myelin structural integrity and function (37). Therefore, ER stress in oligodendrocytes could act as a key mediator of lipid dysregulation, ultimately contributing to myelin instability.

Indeed, transcriptomic analysis revealed that SL is associated with extensive alterations in lipid-associated pathways in oligodendrocytes. Among these, we focused on cholesterol-associated modifications, hypothesizing that they represent a primary driver of SL-related deficits, given cholesterol abundance in myelin and its fundamental role in myelin function (27, 38, 39). Notably, transcripts regulating cholesterol homeostasis and transport were significantly downregulated in the oligodendrocyte transcriptomic profile from SL animals. Specifically, we observed a reduction of Dhcr7, Msmo1, and Mvk, all of which play crucial roles in cholesterol biosynthesis (40). Moreover, Relch and Stard4, key mediators of non-vesicular cholesterol transport (25, 26), were also significantly reduced. Of note, a sustained reduction in Stard4 has been linked to impaired cholesterol transport to the plasma membrane, leading to increased membrane fluidity due to diminished cholesterol content (26). Consistent with these transcriptomic changes, our biochemical analyses confirmed a marked reduction in cholesterol levels associated with a concomitant increase in membrane fluidity within myelin-enriched fractions from SL animals, supporting the presence of lipid compositional imbalance, which could not be explained by a simple reduction in myelin quantity. Given that maintaining high cholesterol content in myelin membranes is critical for preserving proper membrane curvature and ensuring effective electrical insulation (27, 41), SL-induced alterations in cholesterol homeostasis may compromise myelin function. By increasing membrane fluidity, SL could weaken the insulating properties of myelin, disrupt saltatory conduction, and ultimately slow nerve impulse transmission.

In addition to cholesterol modifications, broad lipidomic profiling revealed minimal changes of other lipids, which potentially may contribute to myelin integrity disruption. This approach could not detect cholesterol or its related metabolites because of their poor ionization by the ESI source used in our untargeted analysis. Instead, we observed a trend towards an increase in LPC levels. Although typically absent or present in minimal amounts in myelin, LPC is well-documented for its lipid-disrupting properties, which can compromise myelin stability (42). This increase was accompanied by upregulated transcription of Pla2 (phospholipase A2, Dataset S1), the key enzyme responsible for LPC production (43), and downregulated expression of Lpcat2, which facilitates LPC conversion into phosphatidylcholine (43). These opposing shifts in enzymatic pathways potentially explain the observed LPC accumulation. Furthermore, phospholipase A2 activity is known to be stimulated by increased cellular metabolism and ER stress, both of which are prominent during SL, suggesting a possible mechanistic link between SL-induced metabolic stress and LPC production.

As results of reduced myelin integrity, we observed an approximately 33% average increase in the delay of interhemispheric conduction due to SL, although there was considerable variability among individual animals. This phenomenon was associated with decreased synchronization in the activity of homotopic brain regions. Despite the limitation associated with the use of a common EEG reference, which could have inflated interhemispheric synchronization, the observed changes occurred within the same animals and recording configuration, making the impact of this issue less critical. Notably, the reduction in interhemispheric synchronization was primarily during NREM sleep. One likely explanation is that interhemispheric coupling during NREM depends more directly on intact callosal connections, whereas during wakefulness and REM sleep, activation of arousal systems and elevated neuromodulatory tone promote global cortical integration and help sustain synchrony (44, 45, 46). These mechanisms may mitigate the impact of subtle conduction delays, although the reduction in beta-band coherence observed during wake after SL suggests that fast, frequency-specific interactions remain sensitive to impaired callosal transmission. In contrast, during NREM sleep, when arousal systems are largely inactive, interhemispheric synchronization becomes more vulnerable to small temporal mismatches and relies more heavily on the structural integrity of the CC (47). Further experiments manipulating arousal or neuromodulatory activity could help determine whether enhancing arousal stabilizes interhemispheric communication across vigilance states when callosal connectivity is reduced.

Computational models suggest that even slight alterations in conduction delay can considerably disrupt network activity, leading to shifts in phase-locked interactions among oscillating networks (48). Desynchronized neuronal networks might prompt communication breakdowns between different brain areas, thereby affecting cognitive functions and behavior (14, 49).

In line with this, we observed decreased cognitive and motor performance after SL, which may be attributed to changes in myelin and the resulting disruption in network synchronization (15). Learning deficits as evidenced at the NOR task are common after sleep deprivation (50). While they are generally linked to impaired synaptic plasticity (51), it is possible that a less efficient myelination hinders the integration of information across distributed neural networks, ultimately affecting both the acquisition of new knowledge and the retrieval of previously learned information. Also motor performance exhibited an overall decrease with SL. Given that animals were allowed 24 h to recover from physical fatigue post-sleep restriction, it is unlikely that the observed decrease in motor performance was due to residual fatigue. Instead, it is possible that the integration of information across different brain regions guaranteed by optimal myelination, essential for cognitive tasks, is similarly required for motor tasks involving coordination and precision (13).

Importantly, modifications of both signal propagation and behavior were prevented when the animals were treated with cyclodextrin, a drug that promotes cholesterol re-localization to the myelin membranes (31). This implies that myelin cholesterol deficiency is sufficient to drive SL-associated deficits. It has been demonstrated that the passage of cyclodextrin through the blood brain barrier is limited (52). However, its effectiveness in redistributing cholesterol within brain cells via intraperitoneal or subcutaneous administration has been consistently shown in animal models of Niemann-Pick’s disease, a condition characterized by abnormal intracellular cholesterol accumulation (53, 54). Moreover, since SL can increase the permeability of the blood–brain barrier (55), it is plausible that cyclodextrin passage into the brain might be further enhanced during such conditions, although this possibility has not been directly tested.

It is important to note that, although cholesterol metabolism appears impaired mostly within oligodendrocytes, the observed cholesterol deficits may also originate from mechanisms independent of these cells. Astrocytes are recognized as the primary source of cholesterol in the brain (56), and microglia can also contribute, particularly following demyelination (57). Thus, it is plausible that the effects detected in oligodendrocytes may reflect broader disruptions in cholesterol pathways involving other glial populations, or impairments in the mechanisms responsible for transferring cholesterol to oligodendrocytes.

While our findings demonstrate a beneficial effect of cyclodextrin treatment, it is important to acknowledge that its activity is not restricted to oligodendrocytes. Although a recent study reported that cyclodextrin effects are predominantly exerted on oligodendrocytes compared to other brain cell types (58), we cannot rule out the possibility that its actions on other cell populations also contribute to the observed beneficial effects.

Consistent with previous findings (6), our results also demonstrated that SL significantly reduced OPCs density. As a critical pool for the generation of mature OL, OPCs are indispensable for the dynamic processes of myelination and myelin remodeling (13, 59). This effect was associated to a slight increase in preOL, suggesting a shift toward differentiation driven by wake states, in line with prior observations (6). Notably, disruptions in OPC circadian regulation have been shown to induce sleep disturbances, highlighting a bidirectional relationship between OPC functionality and sleep (60). However, in our study, we did not observe major alterations in circadian gene expression (Dataset S1), possibly because tissue collection occurred at the same time of day for all experimental conditions. This suggests that the observed OPC reduction is a direct consequence of SL rather than an effect of disrupted circadian rhythms. A reduction in OPC availability may compromise myelin plasticity, limiting the ability of myelin sheaths to adapt to evolving neural demands. Consequently, the reduction in myelination observed following SL could partially result from impaired OPC functionality, which hampers the formation of new myelin sheaths required to meet increased neuronal activity imposed by SL. This is consistent with the higher proportion of unmyelinated callosal axons observed in SL rats. However, cyclodextrin treatment was unable to significantly increase the proportion of myelinated axons nor to fully restore OPC numbers in the CC. Despite this, its efficacy in normalizing conduction delays and behavioral outcomes suggests that while OPC reduction and increased unmyelinated axons contribute to SL-induced deficits, they may not be the primary drivers of functional impairments. This points to alternative mechanisms, such as cholesterol homeostasis and myelin integrity, as primary contributors to the observed neurophysiological and behavioral improvements.

This study has some limitations that should be acknowledged. The sleep restriction paradigm employed here represents a relatively severe condition, designed to investigate the consequences of prolonged SL rather than the normal functions of sleep. Therefore, some of the cellular changes observed may reflect adaptive or stress-related responses to sleep disruption. We included a chronic stress condition to account for stress induced by the procedure, but because this protocol could only be applied during a restricted period of the dark phase to avoid disrupting sleep–wake organization, it did not match the intensity or duration of sleep restriction. As a result, although it controlled for part of the stress component, it cannot fully rule out stress as a contributing factor to the observed effects.

In addition, most experiments were conducted in male animals to minimize variability associated with hormonal fluctuations; however, this limits the generalizability of our findings, and future studies should examine whether similar mechanisms occur in females.

In conclusion, our findings establish a close relationship between SL, myelin thinning, delays in signal conduction, and impaired behavioral performance. We identify in the dysregulation of oligodendrocyte cholesterol homeostasis a possible novel mechanistic pathway through which SL disrupts myelin integrity and impairs the efficient propagation of neural signals. These disruptions in myelin function and neural signal transmission are linked to the cognitive and behavioral deficits observed following SL. This work provides a novel framework for understanding the neurobiological underpinnings of the cognitive and behavioral dysfunctions associated with sleep deprivation, suggesting that restoring lipid homeostasis in oligodendrocytes may offer a therapeutic target for mitigating the consequences of SL on brain function.

## Materials and Methods

Detailed experimental procedures are provided in *SI Appendix*.

### Animals.

Male C57BL/6J mice (postnatal day 40 to 60) and Wistar rats (P40–80) were used for all experiments, except for the gene expression analysis, which utilized previously published datasets (GEO GSE48369 and GSE69079). Animals were maintained under a 12:12 h light–dark cycle (lights on 08:00) at 23 ± 1 °C with food and water available ad libitum. All procedures were approved by local ethical committees and conformed to EU and UK animal welfare regulations.

### Sleep Restriction Paradigm.

Sleep deprivation was induced either manually, by introducing novel objects and occasional access to a running wheel, or automatically, using a moving-bar apparatus sweeping the cage floor (4 rpm; Pinnacle Technology). Unless otherwise specified, the SL group underwent continuous restriction for 10 consecutive days, while control sleeping rats (S) experienced identical environmental conditions except that the bar operated for only 3 h/day during the dark phase (4 rpm).

### Cyclodextrin Treatment.

Rats received subcutaneous injections of 2-hydroxypropyl-β-cyclodextrin (2 g/kg; Merck) on days 2, 5, and 8 of the 10-d SL protocol during the dark phase.

### Mild Stress Paradigm.

A separate group of rats was subjected to a 4 h/day a chronic mild stress paradigm for 10 d during the dark phase. Stressors (e.g., wet bedding, food or water removal, cage tilting at 45°, high-frequency rocking at 110 rpm) were applied in random order to avoid habituation.

### EEG/EMG Recordings and Vigilance-State Scoring.

Rats were chronically implanted with epidural screw electrodes over frontal and parietal cortex and neck electromyogram (EMG) wires. Signals were recorded continuously (EEG: 0.1 to 40 Hz; EMG: 10 to 70 Hz; sampling rate 1 kHz, downsampled to 512 Hz) using an OpenEphys system. Sleep–wake states were scored in 4-s epochs according to standard criteria. EEG data were used to quantify the efficacy of the SL protocol and to derive interhemispheric synchronization measures, including Pearson correlation, cross-correlation, magnitude-squared coherence in canonical frequency bands (delta 0.5 to 4 Hz, theta 4 to 9 Hz, sigma 12 to 15 Hz, beta 15 to 25 Hz), and phase-locking values.

### Cortico-Cortical Stimulation.

To assess conduction velocity across the CC, rats were implanted with LFP electrodes in bilateral motor cortex (M1). Under quiet wakefulness, single-pulse stimuli (100 ms, 200 μA) were applied unilaterally, and transcallosal responses were recorded contralaterally. Peak latency, amplitude, and slope of the early negative LFP component (~4 to 5 ms latency) were measured at baseline and 12 h after the final day of sleep/stress manipulation.

### Diffusion MRI and Histology.

After transcardiac perfusion with 4% paraformaldehyde in PBS, rat brains were post-fixed and imaged ex vivo using a 7 T scanner (Bruker BioSpec) with 126 gradient directions (*b* = 0, 4,000, 7,000 s/mm^2^). FA and restricted fraction (RF) maps were generated (DTI and CHARMED model) and analyzed with tract-based spatial statistics (TBSS) using ANTs normalization and threshold-free cluster enhancement. Voxelwise effect sizes in white-matter portions showing significant S-SL differences were expressed as percentage differences relative to controls. Following MRI, sections were stained for MBP and neuronal nuclei (NeuN), whereas oligodendrocyte lineage markers (PDGFRα, CC1, BCAS1) were quantified in a separate set of experimental rats. Images were acquired using epifluorescence or confocal microscopy, and quantitative morphometry was performed with QuPath and Fiji software.

### Electron Microscopy.

CC tissue was processed for EM to measure myelin sheath thickness, g-ratio, axon diameter, and the percentage of unmyelinated axons. Myelin ultrastructure was examined with a Zeiss SIGMA 300 FESEM.

### Transcriptomic Analysis.

Differential expression analyses were performed using publicly available translating ribosome affinity purification (TRAP) datasets from oligodendrocytes and astrocytes (GEO GSE48369 (61) and GSE69079 (62)). Data were normalized using robust multiarray averaging and compared across S and SL conditions using Welch’s *t* test with FDR correction. Functionally enriched pathways were identified using DAVID bioinformatics resources.

### Biochemical and Lipidomic Analyses.

Myelin was purified by successive sucrose gradient centrifugations and hypoosmotic washes. Lipidomic profiling was performed by UHPLC–ESIMS/MS on a Q-Exactive platform using MTBE/MeOH extraction and an internal standard mix covering major lipid classes. Due to the poor ionization efficiency by ESI, cholesterol quantification was performed separately via APCI LC–MS with deuterated cholesterol as internal standard. Membrane fluidity was measured by Laurdan generalized polarization and DPH anisotropy fluorescence assays.

### Corticosterone Measurements.

Rats were briefly anesthetized and decapitated; ~300 μL of blood was collected into lithium-heparin tubes, centrifuged (2,000×*g*, 10 min, 4 °C), and plasma corticosterone quantified using an ELISA kit (Tecan, Switzerland). Samples were diluted 1:4, run in duplicate, and processed in a single assay.

### Behavioral Testing.

Cognitive and motor performance were assessed with the novel object recognition (NOR) and accelerating rotarod tests. In NOR, exploration times were manually scored, and a discrimination index [novel/(novel + familiar)] was computed. In the rotarod task, rats performed 10 trials (10 to 80 rpm over 600 s); the average latency to fall was recorded.

### Statistics.

Statistical tests are described in each section in *SI Appendix*. Source data and statistical test details can be found in Dataset S3.

## Supplementary Material

Appendix 01 (PDF)

Dataset S01 (XLS)

Dataset S02 (XLSX)

Dataset S03 (XLSX)

## Data Availability

Human MRI data were obtained from the preprocessed release (v3.19.0) of the HCP (https://www.humanconnectome.org) (20). Oligodendrocyte and astrocyte transcriptomic data were retrieved from the NCBI GEO database (accessions GSE48369 (61) and GSE69079 (62), respectively). Source data are included in the article and/or *SI Appendix*. All other raw data are available from the corresponding author upon reasonable request. The analysis codes, along with detailed guidelines for reproducing the results presented in this paper, are available on GitHub (https://github.com/BSRLab) (63). All other data are included in the manuscript and/or supporting information.
