# Supplementary material for: Sleep loss induces cholesterol-associated myelin dysfunction
Source: Proc Natl Acad Sci U S A. 2026 Jan 20;123(4):e2523438123. doi: 10.1073/pnas.2523438123 (PMC12846829; doi:10.1073/pnas.2523438123)
Supplement: Supplementary file 1 — Appendix 01 (PDF) [file pnas.2523438123.sapp.pdf]

PNAS

Supporting Information for

Sleep loss induces cholesterol-associated myelin dysfunction.

Reyila Simayi[1,2], Eleonora Ficiarà[1,2], Oluwatomisin Faniyan[2,3], Antonio Cerdán Cerdá[4], Amina Aboufares El Alaoui[2,3], Rosamaria Fiorini[5], Adele Cutignano[6], Fabiana Piscitelli[6], Aroa S. Maroto[4], Alexandra Santos[4], Federico Del Gallo[1,2], Luisa de Vivo[1,2], Silvia De Santis[4], Michele Bellesi[2,3,7]*

1 School of Pharmacy, University of Camerino, Italy.
2 Center for Neuroscience, University of Camerino, Italy.
3 School of Biosciences and Veterinary Medicine, University of Camerino, Italy.
4 Instituto de Neurociencias, Consejo Superior de Investigaciones Científicas - Universidad Miguel Hernández, San Juan de Alicante, Spain.
5 Department of Life and Environmental Sciences, Marche Polytechnic University, Ancona, Italy.
6 Istituto di Chimica Biomolecolare – Consiglio Nazionale delle Ricerche (CNR), Pozzuoli (NA), Italy.
7 School of Physiology, Pharmacology and Neuroscience, University of Bristol, Bristol, UK.

*Corresponding author

Email: michele.bellesi@unicam.it

This PDF file includes:

Supporting text
Figures S1 to S12
Legends for Dataset S1-S3
SI References

Other supporting materials for this manuscript include the following:

Datasets S1 to S3

Supporting Information Text

Animals: C57BL/6J male mice (postnatal day [P] 40-60) and Wistar male rats (P40-80) were used in this study with the exception of gene expression analysis, in which we have referred to a previous database NCBI GEO GSE48369 and GSE69079, obtained in adult (9-10 weeks old) heterozygous 2',3'-cyclicnucleotide3'-phosphodiesterase (CNP)-eGFP-L10a [GSE48369] and aldehyde dehydrogenase 1 family member L1 (ALDH1L1)-eGFP-L10a [GSE69079] bacterial artificial chromosome (BAC) transgenic mice of either sex (1, 2). All animals were under a light/dark cycle 12:12 with lights on at 8 am at 23 ± 1 °C (environmental temperature) and were provided with food and water available *ad libitum* and replaced daily at 8 am. All procedures involving animals adhered to the local Institutional Animal Care and Use Committee and the European Communities Council Directives (2010/63/EU, 542/2023-PR) and to the Animals (Scientific Procedures, P5E96A446) Act 1986 and Amendment Regulations 2012 as outlined in UK law and approved by the University of Bristol Animal Welfare and Ethics Review Board.

Sleep deprivation. In this study, sleep deprivation was induced manually through the introduction of novel objects and occasionally a running wheel or automatically using an apparatus with a moving bar sweeping the cage floor (Pinnacle Technology). For experiments with novel objects, both sleep-deprived and sleeping mice had access to a running wheel and unfamiliar objects during the dark period. Environmental enrichments were removed for the sleep group during the light period, while retained for the sleep-deprived group. Animals were never disturbed while eating or drinking. In experiments with the automatic apparatus, animals were continuously sleep restricted for 10 days (Sleep Loss [SL]) group through the bar movement (speed: 4 rpm). Control sleeping (S group) animals experienced the bar movement only for 3 hours/days (speed: 4 rpm) starting at the beginning of the dark period.

Sleep and wake assessment via video monitoring. In animals used for molecular and morphological analyses, sleep and wake states were monitored using infrared cameras. A custom algorithm, measuring motion, reliably distinguished sleep and wake, achieving about 90% concordance with electroencephalographic (EEG) recordings (3). This motion-based analysis served as a non-invasive alternative to EEG, avoiding inflammation and potential alterations associated with electrode implantation.

Cyclodextrin treatment. 2-hydroxypropyl-β-cyclodextrin (CD, Merck) was dissolved in sterile saline and administered subcutaneously at a concentration of 2g per kg body weight (4). Rats were treated with three injections of CD administered during the dark phase (at 10 am) at day 2, 5, 8 of the 10 days SL experiment.

Mild stress paradigm. Rats were subjected to 4 hours of chronic mild stress paradigm for 10 consecutive days during the dark phase. The procedure involved exposing animals to systematic and repeated mild stressors known to induce behavioral and physiological adaptations (5, 6). Stressors included exposition to no water, no food, wet bedding, tilting of housing cage (45º), and high frequency rocking (110 RPM for 25sec with an interval of 90sec) for 4 hours at the beginning of the dark cycle. These stressors were repeated twice in succession over 10 days.

MRI experiments. Rats were perfused transcardially under anesthesia with 0.9% sodium chloride solution followed by 4% paraformaldehyde in PBS. Brains were extracted and post-fixed in the same fixative solution for 2h. After post-fixation, brains were rinsed in PBS and shipped to the MRI facility, where they were included in a falcon with 3% agarose. MRI was performed on a 7 T scanner (Bruker, BioSpect 70/30, Ettlingen, Germany) featuring maximum gradient intensity of 700 mT/m. Diffusion Weighted Magnetic Resonance Imaging (DW-MRI) data were acquired using a stimulated echo planar imaging diffusion sequence, with 126 uniform distributed gradient directions, b = 0(6), 4000(60) and 7000(60) s/mm^2, diffusion time 15 ms, diffusion duration of 5.5 ms, repetition time (TR) = 5000 ms and echo time (TE) = 28 ms. 30 slices were set up to cover the whole brain with field of view (FOV) = 25×25 mm^2, matrix size = 110 × 110, in-plane resolution = 0.225×0.225 mm^2

and slice thickness = 0.6 mm. In addition, a T2-weighted image was also acquired with the same resolution to facilitate distortion correction and registration to the rat brain MRI atlas. The total acquisition time per subject was 12 hours.
DW-MRI data were corrected for distortions using linear registration, then processed according to standard diffusion tensor analysis with the software ExploreDTI (7), and for CHARMED analysis (8) using custom scripts. As such, maps of fractional anisotropy (FA) and restricted signal fraction (RF) were generated for each subject. FA maps were employed to initialize the first steps of an improved version of the TBSS (9). This version performs the co-registration steps using Ants normalization package (10). We tested for a general linear model comparing SL and S animals, while correcting for multiple comparisons across clusters by using threshold-free cluster enhancement. In portions of the white matter skeleton showing significant group differences in FA, effect size was calculated voxel-wise as the percentage difference between the average FA in the SL group and that in the S group, divided by the average FA in the S group.

Immunofluorescence.
After MR analysis, brains were further cryo-protected in 30% sucrose in PBS, embedded in OCT (Optimal Cutting Temperature compound), frozen to –80°C with isopentane, and finally cut in a cryostat into 30 µm thick serial coronal sections. Coronal slices were rinsed in 1x PBS and antigen retrieval was performed in citrate buffer (10mM Sodium Citrate + 0.05% Tween20 pH 6). The slices were warmed to 80°C for 20 minutes for protein unmasking. The slices were washed with 1x PBS + Triton X-100 at 0.5% for 10 minutes then blocked in the same solution with 5% normal goat serum for 2 hours at room temperature. The slices were then incubated overnight at 4 °C with a primary antibody against myelin basic protein (1:2500; Abcam Cat# ab218011, RRID: AB_2895537). Following primary antibody incubation, the sections were rinsed in PBS and subsequently incubated with the appropriate secondary antibody conjugated to a fluorescent probe (1:500; Invitrogen Cat# A-21428, RRID: AB_141784) for 2 hours at room temperature. After additional washes, sections were treated with DAPI (4′,6-diamidino-2-phenylindole dihydrochloride; 15 mM; Sigma-Aldrich, Madrid, Spain) for 5 min at room temperature to label nuclei. Finally, the sections were mounted onto glass slides, coverslipped, and allowed to dry. Fluorescent images were acquired using a Zeiss Axioscan digital slide scanner, and quantitative analysis of staining intensity was performed using QuPath software. (11). Values were then analyzed with a linear mixed effect model using rat as random variable and condition and brain region as fixed variables. The parameters in the LME models were estimated based on maximum likelihood using the lme4 package in R (12). Likelihood ratio test was used to assess the statistical significance of the brain region and condition effects.
A separate group of experimental rats was perfused with 4 % paraformaldehyde; brain tissues were allowed to post fix in the same fixative for 48h at 4 °C and then was cut on a vibratome in 40 µm coronal sections. Each frontal sections for each rat were rinsed with NGS 8% + 0.1% Triton X-100 for 1h, then simultaneously incubated with a primary antibody anti-platelet-derived growth factor receptor alpha (PDGFR alpha, 1:500, Abcam Cat# ab203491, RRID:AB_2892065) and anti-APC (CC-1, 1:500, Abcam Cat# ab16794, RRID:AB_443473) or anti-brain enriched myelin associated protein 1 (BCAS1, 1:1000, Synaptic System Cat# 445 003, RRID: AB_2864793) in a NGS 5%. After washing with PBS, sections were rinsed with NGS 3% + 0.1% Triton-X for half an hour then incubated with a secondary antibody (1:500) and mounted onto slides for microscopy analysis. Confocal images were acquired at 10× magnification to quantify OPCs, pre-oligodendrocytes, and mature oligodendrocytes within the corpus callosum. Cells were manually annotated and automatically counted using FiJI. Values were then analyzed with a linear mixed effect model using rat as random variable and condition as fixed variables. The parameters in the LME models were estimated based on maximum likelihood using the lme4 package in R (12). Likelihood ratio test was used to assess the statistical significance of the condition effect.

Electron microscopy. Rats were perfused with 2% paraformaldehyde and 2.5% glutaraldehyde. Brains were postfixed in the same fixatives for 1 week at 4 °C and then sliced using a vibratome in 100 µm sagittal sections. Punching of the anterior portion of the corpus callosum was carried out to collect small blocks of tissue, which were subsequently incubated in 1% OsO4/K ferrocyanide for 1 hour, washed in ddH2O, and then incubated in OsO4 2% for 30 min. After washing, tissue

was dehydrated in alcohol and subsequently included in Durcupan resin. Blocks were cut at ~70nm using an ultramicrotome (Leica Ultracut R) and ultrathin sections were collected on copper grids. Next, grids were incubated first in uranyl acetate 2% for 20min in the dark and then in lead citrate (4.5mM) + 200ul NaOH 5M for 5 min after washing in ddH2O. Grids were then imaged with a Zeiss SIGMA 300 FESEM equipped with a STEM detector. G-ratio was calculated by root squaring the ratio between the area of the axonal segment (axonal size) by the area of the fiber segment. In addition to the g-ratio, the axonal segment diameter and the myelin thickness (fiber segment diameter minus axonal diameter)/2 were measured. The number of unmyelinated axons was calculated as a percentage of the total number of axons present in the microscopic field. G-ratio, myelin thickness, and axon diameter were analyzed using a linear mixed effect model using rat as random variable and condition as a fixed variable. The parameters in the LME models were estimated based on maximum likelihood using the lme4 package in R (12) and a likelihood ratio test was used to assess the statistical significance of the condition effect. The density of unmyelinated axons was compared using unpaired t-test.

Human data from HCP repository
We used the pre-processed data provided by the Human Connectome Project (www.humanconnectome.org). The used dataset includes subjects with DW-MRI scans, the latest pre-processing release (v3.19.0), and a complete behavioral assessment, for a total of 185 subjects. All acquisition parameters and processing pipelines are described in detail on the project page.
We fed the pre-processed images to standard diffusion tensor analysis and to CHARMED analysis pipeline (8) using Microstructure Diffusion Toolbox (MDT, https://github.com/robbert-harms/MDT). As such, maps of FA and RF were generated for each subject. The same TBSS approach used in animals was used for the analysis, and skeletonized maps were associated voxel-wise with Pittsburgh Quality Index (PSQI) using a general linear model accounting for age, sex, and intra-cranial brain volume, while correcting for multiple comparisons across clusters by using threshold-free cluster enhancement.

Electroencephalography.
Under deep isoflurane anesthesia (1–1.5% volume), rats were implanted bilaterally for chronic EEG recordings with epidural screw electrodes over the frontal (from bregma: anteroposterior 1 mm, mediolateral, 1 mm) and parietal cortex (anteroposterior, 2 mm; mediolateral, 2 mm) and cerebellum (reference electrode and ground). Two stainless steel wires (diameter 0.4mm) were inserted into neck muscles to record the electromyography (EMG). After recovery, rats were connected by a flexible cable to a commutator and recorded continuously for 2 weeks using an OpenEphys recording system. EEG and EMG signals were filtered (EEG: high-pass filter at 0.1 Hz; low-pass filter at 40 Hz; EMG: high-pass filter at 10 Hz; low-pass filter at 70 Hz). All signals were sampled at 1000 Hz resolution and down sampled at 512 Hz for analysis. Waking, NREM sleep, and REM sleep were manually scored off-line (SleepSign, Kissei COMTEC, Matsumoto, Japan) in 4-s epochs according to standard criteria. NREM and REM episodes were defined as episodes of duration >= 2 epochs (8-s). Repeated measures one-way ANOVA (rANOVA) with factor "day" was performed followed by Dunnett's multiple comparisons post hoc tests (significance level α=0.05) to evaluate statistical difference against baseline. In case of missing values, data were analyzed by fitting mixed-effects models.
To minimize the number of rats that had to be surgically implanted, these animals were also used to carry out the interhemispheric synchronization analysis. In these analyses, we compared baseline data with those obtained during the second day of recovery. We utilized the second recovery day to let the sleep pressure from CSR subside, which typically eased off during the first recovery sleep day. This approach enabled us to compare days (both baseline and the second recovery day) that exhibited analogous sleep-wake patterns.
Interhemispheric synchronization analysis: EEG signals were filtered between 0.15 and 40 Hz by using a FIR (finite impulse response) filter (–6 dB at cutoff frequency, mne.filter function in mne python (13, 14)). Interhemispheric time-domain correlation between EEG amplitude (μV) of the parietal left and right derivation was computed by means of Pearson product-moment correlation coefficient and cross-correlation. Pearson product-moment correlation coefficient for each stage

(NREM, REM and WAKE) was calculated to evaluate how the two signals (EEG channels: parietal left, parietal right) co-vary over time, considering the average of all 4-s epochs of NREM, REM and WAKE.
Pearson coefficient was calculated as

$$R_{xy} = \frac{C_{xy}}{\sqrt{C_{xx}C_{yy}}}$$

where C_{xx} and C_{yy} are the variance and C_{xy} the covariance matrix of two signals x and y respectively (15, 16). Fisher z-transformation was applied to r-values to account for the nonlinearity of correlation coefficients. In addition, we computed mean cross-correlation (16) of parietal left and parietal right signals by averaging the values of all 4-s epochs of NREM, REM and WAKE.
To analyze the coherence in the frequency domains, we used the coherence function defined as the squared cross-spectrum between signals divided by the product of the auto-spectra of each signal (magnitude squared coherence, MSC). Coherence values ranged from 0 to 1, with 1 indicating full correlation (synchronization) between the two signals at a given frequency, while a value near zero suggesting that the signals were unrelated. The coherence value of two signals x and y, $M_{xy}(f)$, was calculated as a function of the spectral densities of signal x, $P_{xx}(f)$, and y, $P_{yy}(f)$, and the cross spectral density of x and y, $P_{xy}(f)$:

$$MSC(f) = M_{xy}(f) = \frac{\left|P_{xy}(f)\right|^2}{P_{xx}(f)P_{yy}(f)}$$

Coherence spectra were calculated between parietal left and parietal right EEG channels for each 4-s epoch considering NREM, REM and WAKE staging. Mean value of coherence was calculated averaging all values of 4-s epochs for each stage respectively. The coherence analysis was performed over specific frequency bands (delta, 0.5–4 Hz; theta, 4–9 Hz; sigma, 12–15 Hz; beta, 15–25 Hz) by averaging the MSC function in the corresponding frequency range.
To quantify the phase synchronization between two EEG channels, we computed the Phase-Locking Value (PLV). PLV is the most commonly used phase interaction measure, evaluating the absolute value of the mean phase difference between the two signals (17). It quantifies the degree to which the phases of two signals are coupled together. PLV ranges from 0 to 1, where 0 indicates no phase locking (completely random phase relationship), and 1 indicates perfect phase locking (both signals have the same phase).
PLV between signals $y_1(t)$ and $y_2(t)$ was computed as follows:

$$z_i(t) = A_i(t)e^{j\varphi_i(t)}$$

The two analytic signals $z_i(t)$, were obtained from $y_i(t)$ (for $i = \{1,2\}$) using the Hilbert transform (HT):

$$z_i(t) = y_i(t) + jHT(y_i(t))$$

Where $HT(y_i(t))$ is the Hilbert transform of $y_i(t)$, which transforms the real signals into a complex representation. Next, we computed the phase difference $\Delta\varphi_i(t)$ between them ($z_i(t)$), with PLV being defined as $PLV(t) = \left|\frac{1}{N}\sum_{n=1}^{N} e^{j\Delta\varphi_i(t)}\right|$, where n indexes the epoch number and N is the total number of epochs (for further details on method see (17, 18)). In our case, the signals were band-pass filtered to delta-theta frequency range (0.5-9 Hz).
Data were expressed as means and standard deviations. Statistical analyses were performed to assess differences between baseline and the second day of recovery within each vigilance state (NREM, REM, WAKE). Differences in correlation coefficients were evaluated using paired t-tests, while differences in MSC and PLV were assessed using the Wilcoxon matched-pairs signed-rank test. Statistical significance was set at $p < 0.05$.

Cortico-cortical stimulation. Under general anesthesia, rats were surgically implanted with LFP electrodes in the primary motor area M1 (AP +2, ML +2) and in the contralateral cortex (AP +2, ML -2). For EEG monitoring, 2 EEG screws were located over the fronto-parietal cortex (AP +4, ML -2 and AP -4, ML -3). The ground and reference were anchored on the cerebellum (Ap -10, ML +2). EMG electrodes were attached to neck muscles. After recovery, they were assigned to sleep loss (SL), sleeping (S), stress, and cyclodextrin (CD) groups. All animals were allowed to acclimatize to the recording set-up placed in a Faraday box (to limit noise during LFP recording) and to the sleep deprivation chamber for 2 hours before the recording. After acclimatization sessions, three sessions

of recordings were performed: 1) a pre-stimulation session to test the input-output signal, signal to noise ratio of the inner/outer channels of the LFP electrodes and to determine optimal stimulation intensity for each animal; 2) baseline before the sleep manipulation experiment (10 days of S, SL, Stress, or SL+CD); 3) Post experiment session after the end of the sleep manipulation experiment. Electrical stimuli were delivered with an isolated stimulator (ISO-flex; A.M.P.I., Israel) as square-wave pulses (100 ms, 200 µA) and recorded with an OpenEphys acquisition system with a sampling rate of 30KHz. All stimulation occurred in quiet wake. To avoid confounding effects of behavioral state on evoked responses, we carefully monitored the behavior of each rat using LFPs, muscle activity and direct visual observation, and recorded the evoked LFPs under standardized conditions of quiet wakefulness and at the same time of day. All rats were recorded prior (baseline session) and after the sleep manipulation (post session). Notably, post stimulation sessions occurred always 12h after the end of the sleep manipulation to allow the rats to properly recover from the physical activity associated with the sleep restriction procedure. Pre-stimulation session consisted in 20 stimulations/intensities (ranging between 1-60 volts, with a train interval of 15 mins) to carry out a stimulation-response curve used for detecting optimal stimulation intensity. For the baseline and post experiment sessions, 100 pulses (100 ms, 200 µA) at the selected intensity were delivered and the early component of the LFP evoked response was recorded contralaterally. The side of stimulation (right or left) was randomly assigned to the animals. We focused on the transcallosal response because the corpus callosum consists of a distinct, isolated and homogenous bundle of myelinated excitatory fibers, and thus the early monosynaptic component of the evoked response can be easily identified. The response consisted of a depth-negative wave with latency to the peak of ~ 4-5 ms. Latency was stable within each session and showed minimal (<1ms) variability. The peak latency, amplitude, and slope of the evoked LFP response were estimated in custom-made MATLAB routine. The slope of the component was computed as mean first derivative of the first down-going segment. Repeated measures ANOVA with time as within factor and group as a between factor was used to evaluate differences between baseline and post experiment sessions. Šídák's multiple comparisons test was used as a post-hoc test. After the last stimulation session, rats were perfused transcardially under anesthesia for histological evaluation of LFP electrodes position.

<u>Gene expression analysis:</u> we used the array data available at NCBI GEO database (GSE48369 (1) and GSE69079 (2)) to perform gene expression analysis of forebrain samples collected from S and SL mice. Samples of this database were collected using the genetically targeted translating ribosome affinity purification (TRAP) methodology from BAC transgenic mice expressing EGFP tagged ribosomal protein L10a in oligodendrocytes (GSE48369) or astrocytes (GSE69079). This method permits to study the expression of mRNAs attached to ribosomes on their way to become proteins, thus providing a better functional overview than traditional methods based on estimates of whole RNA. For each animal, one forebrain sample was immediately processed for immunoprecipitation to isolate either oligodendrocytes or astrocytes mRNAs. The immunoprecipitated fraction constituted the bound sample (IP), which was enriched for the targeted cell type, while the remaining fraction formed the unbound sample (UB), enriched in all other brain cell types, including neurons and other glial cells. Both IP and UB samples were subsequently processed for RNA extraction and analyzed using Affymetrix GeneChip Mouse Genome 430 2.0 arrays. In the present study, we used array data obtained only from samples of S and SL mice for the oligodendrocyte (IP and UB) and astrocyte (IP only) datasets. Data were normalized within each behavioral state group using Robust Multiarray Average. To identify transcripts that were differentially expressed across S and SL, comparisons were carried out using Welch's t test with Benjamini and Hochberg false discovery rate (FDR) multiple-test correction. The lists of differentially expressed genes was submitted to the DAVID (Database for Annotation, Visualization and Integrated Discovery) bioinformatics database for functional annotation (http://david.abcc.ncifcrf.gov/)(19). The background list used in the program included all the genes used for the differential expression analysis.

<u>Myelin tissue collection and preparation.</u> Mice were sacrificed with cervical dislocation, while rats were sacrificed with decapitation under general anesthesia with isoflurane. Then their brains were quicky removed and snap frozen. To obtain myelin preparations we followed a modified version of

the LaRocca's protocol, which provided an optimal balance between myelin purity and the preservation of myelin multilayered structure (20). Briefly, brain tissue was weighted and then homogenized on ice using a glass potter containing in 0.3 M sucrose solution with 20mMTris·Cl buffer (pH 7.45), 1mM EDTA, 1mM DTT, 100 µM phenylmethylsulfonylfluoride (PMSF), 10 µg/ml leupeptin, and a mixture of anti-proteolytic compounds (cOmplete Tablets, Roche). The homogenate was layered over 0.83 M sucrose solution and centrifuged 35 min at 75,000×g. The band of crude myelin membranes formed at the 0.3 M /0.83 M sucrose interface was collected, and after washing out the sucrose with hypotonic buffer, was subjected two times to a cycle of hypoosmotic shock and low-speed centrifugation (15 min at 12,000 × g) to remove cytoplasmic and microsomal contaminants. Then myelin was further purified with a repetition of the first density gradient centrifugation and a cycle of hypoosmotic shock and low-speed centrifugation. Finally, a highly purified myelin fraction was prepared by a third density gradient centrifugation. However, in this step myelin was resuspended in 0.83 M sucrose, and the 0.83 M sucrose solution was laid over with 0.30 M sucrose. The myelin fraction at the interface was subjected to a final hypoosmotic shock cycle, collected, and resuspend in 500 µl of Tris·Cl buffer. Protein concentration was assessed using a spectrophotometer.

LC-MS/MS lipidomic analysis. Highly-purified myelin fractions (200µl) were extracted with MTBE/MeOH/H2O (10:3:2.5) according to Matyash et al. (21). Briefly, to each sample were added MeOH (290 µl) and the IS pool (10 µl MeOH). After vortexing 1 ml of MTBE was added, and the sample sonicated and vortexed again, was allowed to extract at 10°C under shaking. After 1 hour, 250 µl of milliQ water were added to induce phase separation. The sample was then centrifuged at 10,000 g for 10 min at 4°C and the upper phase was recovered. The extraction was repeated by adding 300 µl MTBE and the organic extract recombined, dried under nitrogen stream and vacuum, and stored at -80°C until analyses. For LCMS, samples were reconstituted in 200 µl MeOH/isopropanol (1:1).
Chromatographic separations were achieved on Infinity 1290 UHPLC System (Agilent Technologies, Santa Clara, CA, USA), equipped with a Kinetex Biphenyl 2.6 µm, 150 x 2.1 mm column, (Phenomenex, Castel Maggiore, Bologna, Italy) according to (22). Briefly, eluent A was acetonitrile/H_2O 60:40, 10 mM ammonium formate, 0.1% FA and eluent B: isopropanol/acetonitrile 90:10, 2 mM ammonium formate, 0.1% FA. All solvents were LC-MS grade. The elution program consisted of a gradient from 20 to 40% B in 6.5 min, then to 50% B up to 13 min, reaching 90% B at min 16, holding for 1 min and returning to 20% B in 1 min. A post run equilibration step of 5 min was included prior to each analysis. Column temperature was set at 40°C. Flow rate was 0.3 ml/min. The injection volume was 5 µl and the autosampler was maintained at 10°C.
MS analyses were carried out on Q-Exactive Hybrid Quadrupole-Orbitrap mass spectrometer (Thermo Scientific, San Jose, CA, USA) equipped with a HESI source. Source parameters were as follows: spray voltage positive polarity 3.2 kV, negative polarity 3.0 kV, Capillary temperature 320°C, S-lens RF level 55, Auxiliary gas temperature 350°C, Sheath gas flow rate 60, Auxiliary gas flow Rate 35. Full MS scans were acquired in the range 150–1800 m/z at 70000 of mass resolution, AGC Target 1e6, Acquisition time 100ms. For MS/MS analysis a data dependent ddMS2 Top10 method was used; Mass Resolution was 17500, AGC Target 1e5, Acquisition Time 75 ms. Mass fragmentation was obtained with a stepped normalized energy (NCE) of 16–20-30 and 20–40 in positive and negative ionization mode, respectively. A pool of commercial and in-house synthesized standards was used as Internal Standard mix for quantitative purposes. After preliminary experiments run to assess the appropriate concentration, the customized mix included the following (final concentration in parentheses): Triacylglycerol 17:0/17:0/17:0 (1 µg/ml), Diacylglycerol 15:0/18:1 (0.1 µg/ml), Phosphatidyl choline 10:0/10:0 (2 µg/ml), Phosphatidyl choline 15:0/18:1 (2 µg/ml), Phosphatidyl ethanolamine 17:0/17:0 (2 µg/ml), Phosphatidyl serine 17:0/17:0 (2 µg/ml), Phosphatidyl inositol 15:0/18:1 (2 µg/ml), Phosphatidyl glycerol 17:0/17:0 (2 µg/ml), Lysophosphatidyl choline 17:0 (0.2 µg/ml), Lysophosphatidyl ethanolamine 17:1 (0.2 µg/ml), Ceramide d18:1/17:0 (0.5 µg/ml), Glucosyl ceramide d18:1/17:0 (0.2 µg/ml), Plasmalogen 18:1d9 (2 µg/ml), Sphingomyelin d18:1/17:0 (1 µg/ml), Sulfogalactosyl ceramide d18:1/17:0 (0.5 µg/ml), MGDG 19:0/19:0 (1 µg/ml). All standards were purchased from Avanti Polar Lipids. except MGDG which was synthetized in house as reported in (23).

Each analysis was run in triplicate. Raw LC-MS/MS data were processed by Xcalibur software (Thermo Scientific, version 3.1.66.10); lipid species were identified with the support of LipidSearch software (Thermo Scientific, version 4.1.30). A tolerance of 5 ppm was set for Precursor Ion and 10 ppm for Product Ion. The m-score threshold was set to 5. The lipid identification lists were aligned for control and treated samples and compared by their lipid class and lipid species levels using a retention time tolerance of +/- 0.25 min. The main grade was set to A, B and C for all lipid classes. All data were manually double checked. Absolute quantitative data were reported as µg lipid/ml myelin sample. Data were normalized using median-center method. Differentially expressed analysis was performed to find significant lipid species. Sample expression data were analyzed with unpaired t-test with p-value adjusted by Benjamini-Hochberg procedure (FDR=5%).

<u>LC-MS analysis of cholesterol</u>. Highly-purified myelin fractions (200µl) were extracted with a modified Bligh and Dyer protocol (24). Briefly, samples were sonicated and then extracted with chloroform/methanol (2:1, v/v) containing internal deuterated standard for cholesterol quantification by isotope dilution (2 µg/ml for d7 cholesterol, Merck). Organic phases were collected and dried down under nitrogen. Then lipid extracts were analyzed by liquid chromatography-atmospheric pressure chemical ionization-single quadrupole mass spectrometry (LC-MS2020 Shimadzu). Briefly, using APCI positive ionization, cholesterol was acquired in SIM mode with a m/z of 369.5 and 376.5. MS parameters were the following: acquisition time 0-15, event time 0.5 sec, detector voltage 1.7 kV, interface temperature 400 °C, DL temperature 250 °C, heat block 230 °C, nebulizing gas flow 3 L/min and drying gas 5 L/min. A Phenomenex Kinetex C18 (5 µm x 4.6 mm x 150 mm) column was used for isocratic elution utilizing 95% Mobile Phase B for 15 minutes at a flow rate of 0.5 mL/min. Cholesterol eluted off the column with a retention time of 5.54 min. The column temperature was 40°C and an injection volume of 10 µL was used. Mobile Phase A consisted of 0.1% formic acid in DiH2O while Mobile Phase B contained 0.1% formic acid in acetonitrile. Endogenous levels of cholesterol were calculated based on their area ratio with the internal deuterated standard signal areas and normalized to ml of myelin, protein content, or brain dry weight. Differences between groups were analyzed with unpaired t-test and one-way ANOVA followed by Tukey's multiple comparisons test in case of three groups.

<u>Membrane fluidity.</u> Physico-chemical studies of highly purified myelin fractions were performed by using two fluorophores: 2,dimethylamino-lauroyl-naphtalene (Laurdan), located at hydrophobic hydrophilic interface of the membrane, and 1,6 diphenyl 1,3,5 hexatriene (DPH), incorporated at different levels of the membrane hydrophobic core. The quantum yields of fluorescence of both probes are virtually zero in aqueous solutions, while are quite high in membranes.
DPH fluorescent anisotropy is widely used to study the organization and dynamics of the internal regions in membranes (25). This parameter reflects the bilayer's resistance to molecular rotational and translational movements, offering information on lipid packing in the membrane.
The fluorescence anisotropy was calculated by using the following equation:
$rs = (I_{\parallel} - I_{\perp} x g)/(I_{\parallel} + 2I_{\perp} x g)$ where g is an instrumental correction factor, $I_{\parallel}$ and $I_{\perp}$ are the emission intensities with the polarizers parallel and perpendicular to the direction of the polarized exciting light, respectively.
Laurdan fluorescence excitation and emission spectra are affected by the amount of water (polarity) and by the motion of water molecules (dipolar relaxation of water molecules) close to the fluorescent moiety. Spectroscopic data were used to calculate the excitation and emission generalized polarization spectra, which provide information about the lipid packing (fluidity) of the membrane and the phospholipid phase (26). Laurdan excitation GP (Ex GP) and emission (Em GP) spectra have been calculated as follows:
Ex GP = $(I_{440}-I_{490})/(I_{440}+I_{490})$ where I_{440} and I_{490} are the intensities at each excitation wavelength, from 320 to 420 nm, obtained using a fixed emission wavelength of 440 and 490 nm, respectively; Em GP = $(I_{390}-I_{360})/(I_{390}+I_{360})$ where I_{390} and I_{360} are the intensities at each emission wavelength, from 420 to 550 nm, obtained using a fixed excitation wavelength of 390 and 360 nm, respectively. Stock solutions of Laurdan (6-dodecanoyl-2-dimethylamine-naphthalene) in ethanol and DPH (1,6-diphenyl-1,3,5-hexatriene) in tetrahydrofuran were added to myelin membranes at final probes concentration of 1 µM; each suspension was incubated in the dark, at 37 ◦C for 2 hours prior to use. The fluorescence measurements have been performed at 37°C with

a computer-controlled PerkinElmer LS55 spectrofluorometer. The background fluorescence of the samples was checked prior to each measurement and was negligible when the probes were added. Differences between groups were analyzed with unpaired t-test for DPH and 2-way ANOVA with wavelength and conditions as factors for Laurdan measurements.

BODIPY-cholesterol assessment. *BODIPY-cholesterol fluorescent in fresh tissue:* we used BODIPY-cholesterol to detect cholesterol levels in isolated myelin enriched samples prepared as previously described. BODIPY-cholesterol is a fluorescently labeled cholesterol analog used for quantifying and visualizing the expression level in tissues, homogenates, and live cells. We used 100 µl of myelin-enriched preparations from each group of samples and incubated with 20 µg/ml BODIPY-cholesterol for an hour at 37 °C. After incubation, samples were quickly centrifuged at 12,000 g for 10 minutes and pellets (corresponding to the fraction bound to BODIPY-cholesterol) and supernatants (corresponding to the fraction unbound to BODIPY-cholesterol) were collected. Pellets were then resuspended in 50 µl double distilled water. Fluorescence from bound and unbound fractions was then measured using a plate reader (excitation: 482nm; emission: 515 nm -Tecan i-control plate reader). For each sample, we read also a blank with no BODIPY-cholesterol as a negative control.

BODIPY-cholesterol fluorescence in fixed tissue. Rats were perfused transcardially under anesthesia with 0.9% sodium chloride solution followed by 4% paraformaldehyde in PBS. Brains were post-fixed for 48 hours and then sliced into 40 µm sections using a vibratome.

After blocking with normal goat serum (NGS) 10% with 0.05%TritionX-100, frontal sections were incubated with BODIPY-cholesterol (TopFluor, Avanti Polar Lipids) at 8 µg/ml in association with myelin basic protein antibodies (MBP, 1:1000, Abcam Cat# ab65988 RRID: AB 1139419) at room temperature (2h) and then at 4°C overnight. After washing with PBS, sections were incubated with secondary antibodies (1:1000) and mounted onto slides for microscopy analysis. Confocal fields (3 per section, 2 sections per rat) were acquired in the corpus callosum. MBP staining was used to narrow the analysis on myelin enriched regions. Images contained mostly myelinated fibres and sporadic cells that were outlined and not included in the analysis. To estimate cholesterol levels, mean 488 nm fluorescence intensity was calculated for each image using FiJI.

To estimate BODIPY-cholesterol levels in neurons and astrocytes, frontal sections were first incubated with a blocking buffer (bovine serum albumin 3% with 0.3%TritionX-100 for NEUronal Nuclei [NeuN], NGS 10% with 0.05%TritionX-100 for Glial fibrillary acidic protein [GFAP]) for 1 hour and then with BODIPY-cholesterol (TopFluor, Avanti Polar Lipids) at 8 µg/ml in association with antibodies against NeuN (1:200, Synaptic system Cat# 266011, RRID:AB_2713971) or GFAP (1:500, Sigma, Cat# G3893, RRID:AB_477010) overnight at 4°C. After washing with PBS, sections were incubated with secondary antibodies (1:1000) and mounted onto slides for microscopy analysis. Confocal fields (3 per section, 2 sections per rat) were acquired in the lower layers of the frontal cortex. To estimate cholesterol levels in neurons and astrocytes, somas of NeuN and GFAP positive cells were manually segmented and mean 488 nm fluorescence intensity referring to BODIPY-cholesterol was measured within each individual cell using FiJI.

For experiments on fresh tissue, values were analyzed with ANOVA followed by Tukey's multiple comparisons test. For experiments in fixed tissue, a linear mixed effect model using rat as random variable and condition as a fixed variable. The parameters in the LME models were estimated based on maximum likelihood using the lme4 package in R (12). Likelihood ratio test was used to assess the statistical significance of the condition effect.

Corticosterone measurements. S, SL, and Stress rats were briefly anesthetized with isoflurane and decapitated using a guillotine. Approximately 300 µL of blood was collected from the chest cavity of each rat into lithium-heparin-coated Microvette tubes (Sarstedt, Germany) and centrifuged at 2000 × g for 10 min at 4 °C to obtain plasma. Plasma corticosterone levels were measured using a Corticosterone ELISA kit (#re52211, Tecan, Switzerland). Samples were diluted 1:4 in assay diluent and analyzed in duplicate within a single assay following the manufacturer's instructions. Values were analyzed using one-way ANOVA.

Novel object recognition test. Before starting the experimental set up, all rats were handled briefly three times during the 10-day sleep restriction period. The experiment began with a one-day

habituation, starting at 9 am. This included 30 minutes of habituation to the experimental room, followed by 8 minutes in an empty arena (45 x 45 x 60 cm). On the test day, spatial cues were attached to the walls of the room. The test day started with 20 minutes of habituation to the experimental room, followed by 10 minutes of familiarization with identical objects, after which the rats were removed from the arena and returned to their home cages. After three and a half hours, a 10-minute testing phase was conducted, introducing both a novel object and a familiar object previously seen by the rats. To have optimal and perfect experimental conditions, each time that the arenas and objects were thoroughly cleaned with soap and 70% ethanol, followed by another soap wash to remove any residual odors from previous rats that performed the test. In addition, the location of the novel object was changed at least once to prevent the rats from developing spatial preferences. Each trial was recorded using EthoVision XT software, and videos were analyzed manually with a stopwatch. Exploration behavior was scored based on the following criteria: rats were considered as exploring if they directed their nose toward the object within a 2 cm distance, or if they sit on top and had its nose directed toward the object. Touching the objects with their paws without directing the nose towards the object as well as sitting on top of the object while looking at surrounding environment was not considered exploration. During analysis, chewing was excluded from the total exploration time, as chewing does not qualify as exploratory behavior(27). The discrimination index was calculated by dividing the time that the animal explored the new object by the total time that the animal interacted with either object. Data were analyzed using one-way ANOVA followed by Tukey's multiple comparisons test.

<u>Rotarod experiment</u>. We used an accelerating rotarod system from Ugo Basile SRL. Rats were placed onto a stationary rod and acceleration began. The acceleration profile was linear (starting speed 10 rpm; final speed: 80 rpm; time to accelerate from 10 to 80 rpm: 600 sec). Times when rats fell off the rod were automatically recorded. Sessions included 10 trials for each rat. Notably, to avoid the confounding effect of physical fatigue on the motor performance evaluation, SL rats were given 12 hours of rest during the light cycle before testing: during this period the animals were allowed to sleep and rest ad libitum. To reduce the procedural stress, all rats were manually handled by the same operator that performed the behavioral test and briefly (5 min) exposed to the rotarod apparatus in off-mode for at least two weeks prior to the test. A motor performance score was calculated for each rat by averaging the scores of each trial. Data were analyzed using one-way ANOVA followed by Tukey's multiple comparisons test.

<u>Statistics</u>: statistical methods used to derive significance are described in each section of the previous paragraphs and summarized in Dataset 3. Computational and statistical analyses were performed by using Python 3.9, Matlab (version 2024*a*, The MathWorks, Inc.), R, and GraphPad Prism 9.5.1. Notably, for our EM and immunohistochemical analyses, in which we collected repeated measurements from the same experimental units (i.e., individual animals), we employed linear mixed-effects (LME) models with subjects (animals) included as random effects. This approach appropriately accounted for within subject pseudo-replications, which would otherwise violate the assumption of independence required by many classical statistical methods.
Specifically, we specified a model structure with the animal as a random intercept, allowing us to account for individual baseline differences and control for the non-independence of repeated observations from the same animal. Parameter estimation was performed using the lmer() function from the lme4 package in R. Model assumptions, including the normality and homoscedasticity of residuals, were verified through diagnostic plots.
To evaluate the significance of the fixed effect of condition, we conducted a likelihood ratio test comparing the full model to a reduced model without the condition term. In this work, we used LME of the form:

$$Y_{i,j} = \beta_0 + b_j + \sum_{k=i}^{K} \beta_k X_{i,j,k} + \epsilon_{i,j}$$

$$b_{0,j} \sim N(0, \sigma^2_{animal})$$

$$\epsilon_{i,j} \sim N(0, \sigma^2)$$

$Y_{i,j}$ is the response variable (e.g., g-ratios, myelin thickness, OPC, PreOL, and OL density values, etc.) of the ith observation from the jth animal. The values $b_{0,j}$ were the random intercepts for each animal. The random intercepts were assumed to be normally distributed, with mean zero and constant variance. The β values corresponded to the fixed effects in the model, β_0 is the model intercept, and β_k the effect of the kth explanatory variable. The design matrix X contained the values for each explanatory variable. The residuals $\epsilon_{i,j}$ were checked to be normally distributed and with constant variance.

Figures

Figure S1.

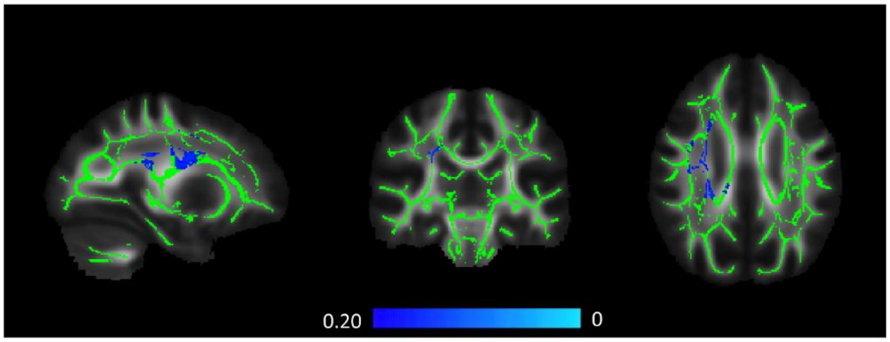

Correlation between restricted fraction (FR) and Pittsburgh sleep quality index (PSQI) score. Blue/light blue represent the white-matter regions, from tract-based spatial statistics (TBSS) with negative correlation between FR and PSQI (n = 185). Correlational analyses were conducted using randomize threshold-free cluster enhancement (TFCE), and family-wise corrected for multiple comparisons. The results are shown in overlay on the mean FA template and the mean FA skeleton (green), calculated from all the subjects. Axial and coronal sections are shown with the right hemisphere on the left; sagittal section is shown with posterior on the left.

Figure S2.

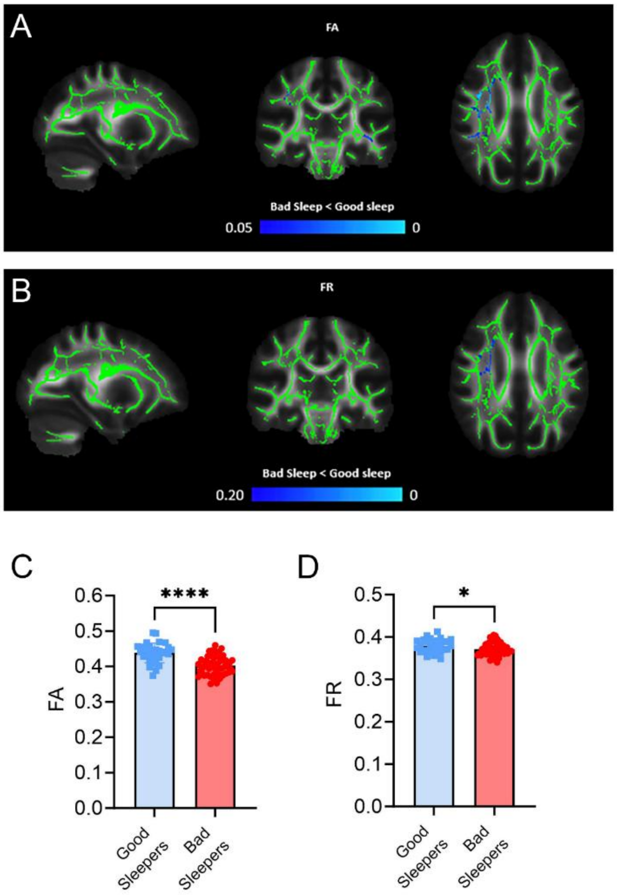

Comparison of WM integrity between poor and good sleepers. (A and B) Fractional anisotropy (FA, A) and restricted fraction (FR, B) differences between poor (PSQI > 5, n = 51) and good (PSQI < 3, n = 30) sleepers. Blue/light blue represent the white-matter regions, from tract-based spatial statistics (TBSS), with significantly reduced FA (A) and a trend toward the reduction for FR (B) in the poor sleep group. Correlational analyses were conducted using randomize threshold-free cluster enhancement (TFCE), and family-wise corrected for multiple comparisons. The results are shown in overlay on the mean FA template and the mean FA skeleton (green), calculated from all the subjects. Axial and coronal sections are shown with the right hemisphere on the left; sagittal section is shown with posterior on the left. (C) Mean FA between groups in regions where FA was significantly different in TBSS analysis. Statistical significance was addressed with unpaired t-test. ****P<0.0001. (D) Mean FR between groups in regions where FA was significant in TBSS analysis. Statistical significance was addressed with unpaired t-test. *P<0.05.

Figure S3

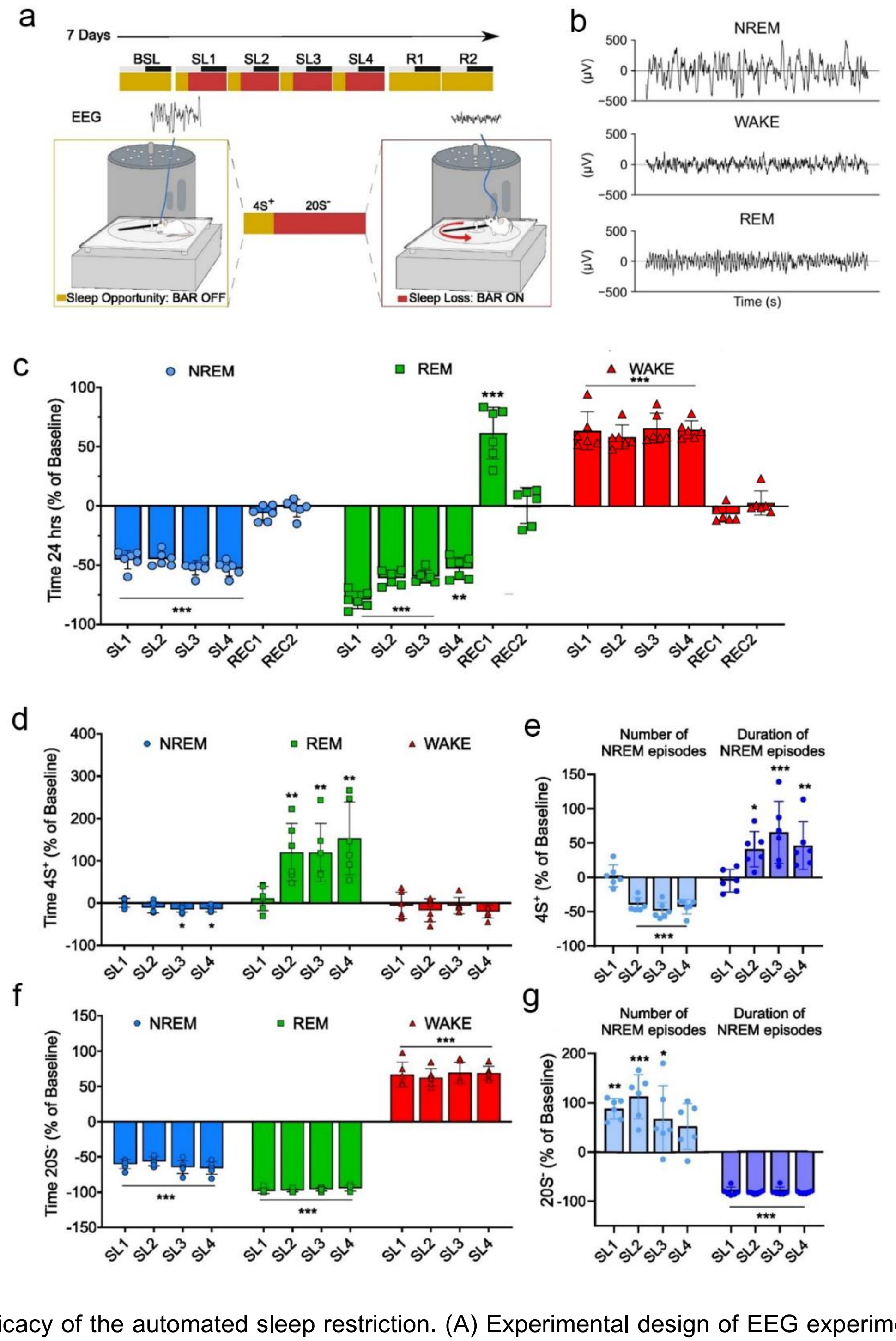

Efficacy of the automated sleep restriction. (A) Experimental design of EEG experiments (n = 6). BSL = baseline (1 day), SL = sleep loss (4 days), R = recovery (2 days, REC 1-2) from SL. 4S+ (in yellow) indicates 4-hour sleep opportunity, 20S- (in red) indicates sleep restriction. (B) Examples of EEG traces in WAKE and NREM and REM sleep. (C) Time spent in NREM (blue) and REM (green) sleep was reduced (rANOVA, for NREM Fday (2.685, 13.43) = 116, P<0.0001, for REM Fday (2.438, 12.19) = 222.5, P<0.0001), while it increased in wake (red, Fday (2.358, 11.79) = 188.6, p<0.0001). The duration of REM sleep increased during REC1 (P = 0.0006). (D) During

4S+ the time spent in NREM (blue) sleep did not change in SL1 and 2, while it slightly decreased in SL3 and SL4 (rANOVA, for NREM Fday (2.097,10.48) = 7.040, P = 0.0019). The time spent in REM (green) sleep increased in SL2, SL3, and SL4 (Fday (2.212,11.06) = 34.48, p<0.0001). (E) Reduced number but larger mean duration of NREM episodes during $4S^+$ in SL2, SL3, SL4 (number: rANOVA, F(4, 20) = 47.79, P<0.0001); duration: rANOVA, (4, 20) = 11.46, P<0.0001). (F) During the 20S- the time spent in NREM (blue) and REM (green) sleep was reduced, while the time spent in wake (red) was increased for SL1-4 (rANOVA, for NREM Fday (2.303, 11.52) = 70.70, P<0.0001, for REM Fday (1.171,5.857) = 137.7, p<0.0001, for wake Fday (2.491,12.46) = 111.7, P<0.0001). (G) During $20S^-$, NREM episodes increased in SL1, SL2, SL3 (rANOVA, F (4, 20) = 7.233, P = 0.0009) while their related mean duration was shorter for all SL1-4 (rANOVA, F (4, 20) = 111.0, P<0.0001). (C-G) values are expressed as % of the baseline. *P<0.05. **P<0.01. ***P<0.001.

Figure S4

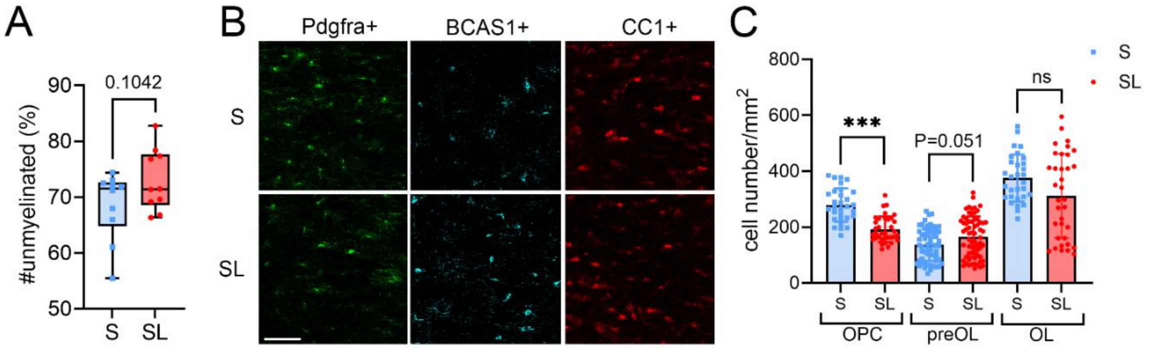

Effects of S and SL on unmyelinated axons, OPCs, preOligodendrocytes (preOL), and Oligodendrocytes (OL) densities in the corpus callosum. (A) Percentage of unmyelinated axons in S (n = 10 EM images, 3 rats) and SL (n = 10 EM images, 3 rats). (B) Representative images of Pdgfra+ (OPCs, green), BCAS1+ (preOL, cyan), and CC1+ (OL, red) cells from a S and SL rat. Scale bar: 50µm. (C) Quantification of OPC, preOL, and OL densities in S (OPC: n = 30, preOL: n = 60, OL: n = 30; 6 rats) and SL (OPC: n = 35, preOL: n = 66, OL: n = 35; 7 rats) rats. ***P<0.001. ns: not significant.

Figure S5

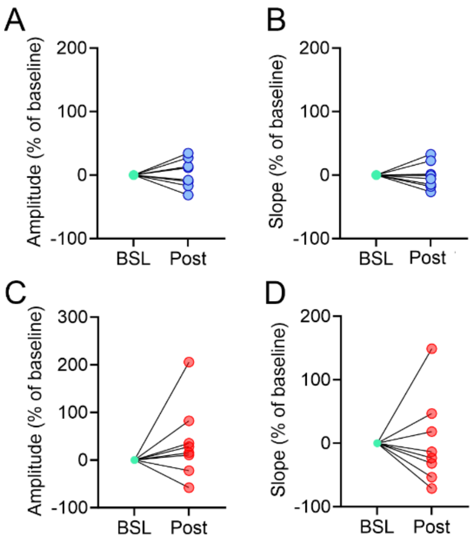

Effects of S and SL on amplitude and slope of the cortico-cortical transcallosal evoked responses. (A and B) Amplitude (A) and slope (B) of the early component negative peak for S individual rats (n = 8). (C and D) Amplitude (C) and slope (D) of the early component negative peak for SL individual rats (n = 8).

Figure S6

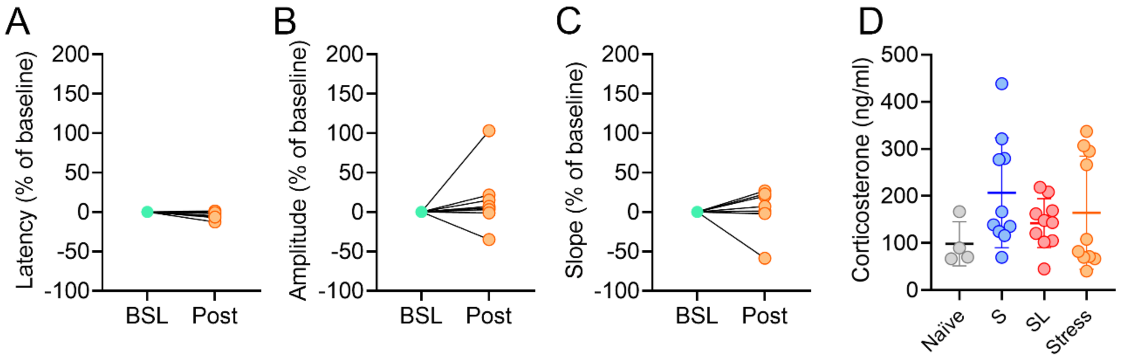

Effects of S, SL, and Stress on latency, amplitude, and slope of the cortico-cortical transcallosal evoked responses and on plasma corticosterone levels. (A, B, and C) Latency (A), amplitude (B), and slope (C) of the early component negative peak for Stress individual rats (n = 8). (E and F) Amplitude (E) and slope (F) of the early component negative peak for Stress individual rats (n = 8). (D) Plasma corticosterone levels in Naïve, S, SL, and Stress rats. Naïve rats were not subjected to any experimental manipulation.

Figure S7

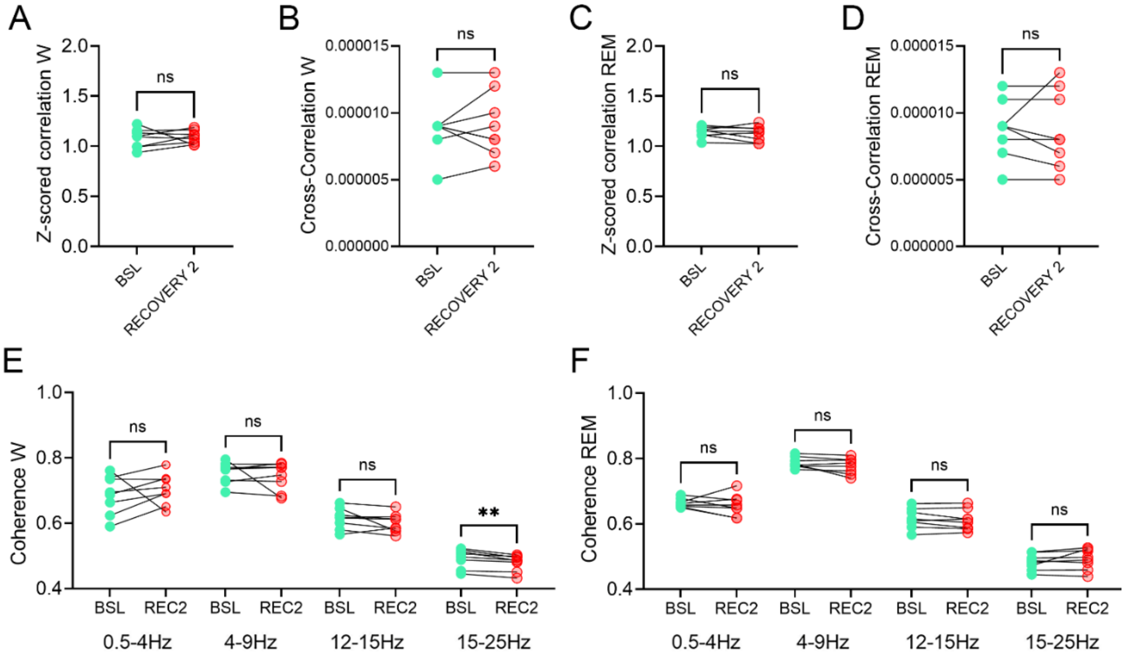

Interhemispheric synchronization in wake and REM sleep. (A-D) Interhemispheric z-scored correlation and cross-correlation values for SL rats (n = 8) at baseline and on the second day of recovery after sleep restriction (Recovery 2). Panels (A and B) show data for wake (W), and panels (C and D) for REM sleep. (E and F) Interhemispheric coherence during wake (E) and REM sleep (F) at baseline and Recovery 2 (REC2). **P < 0.01.

Figure S8

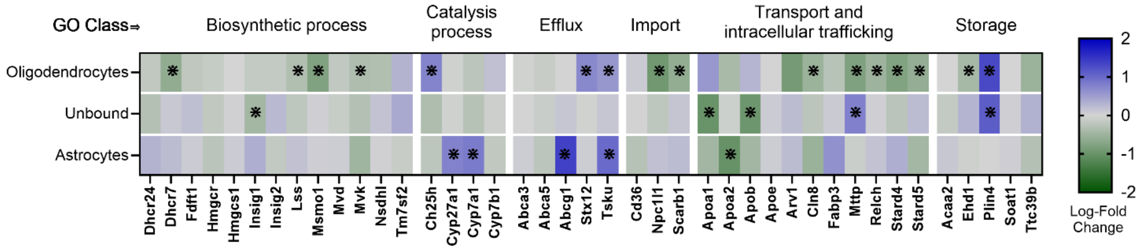

Expression changes of major cholesterol pathways in oligodendrocytes, unbound samples, and astrocytes. Gene expression changes in the cholesterol pathway according to GO biological process annotation classes from the oligodendrocyte dataset, unbound dataset (all brain cells except oligodendrocytes), and astrocytes. Values expressed as log-fold changes compared to S are shown in the heat map. Asterisks indicate significant changes.

Figure S9

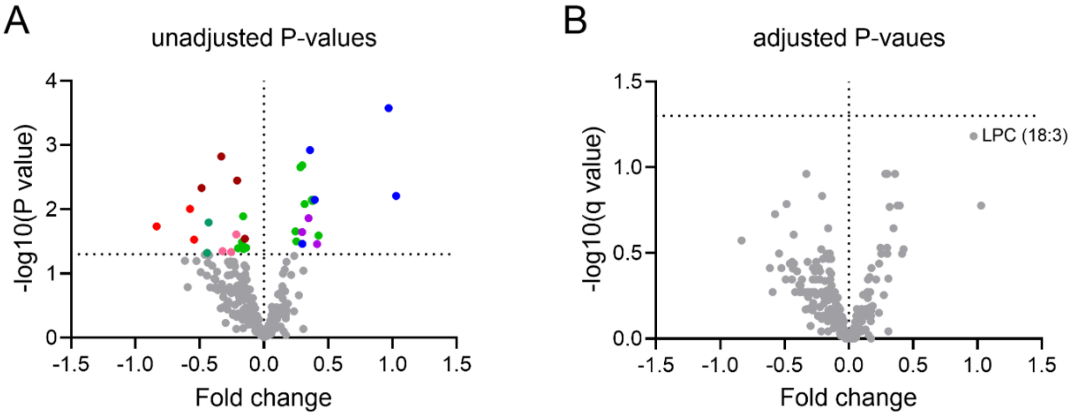

Effects of S and SL on other lipid species. (A and B) Volcano plots of lipid species showing fold-change versus statistical significance before (A) and after (B) multiple-comparison correction using the false discovery rate (FDR, 5%). Lipid classes are color-coded: lysophospholipids (blue), glycerophospholipids (green), acylcarnitines (purple), sulfatides (brown), triglycerides (red), and ceramides (pink). The horizontal dashed line marks the significance threshold.

Figure S10

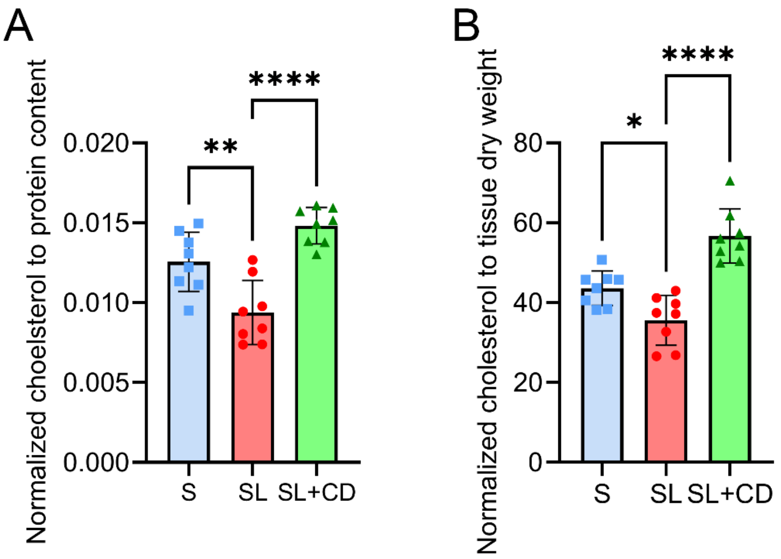

Normalized cholesterol levels in myelin purified membranes. (A and B) Cholesterol levels normalized to protein content (A) and tissue dry weight (B). n = 8 for all groups, *P<0.05, **P<0.01, ****P<0.0001.

Figure S11

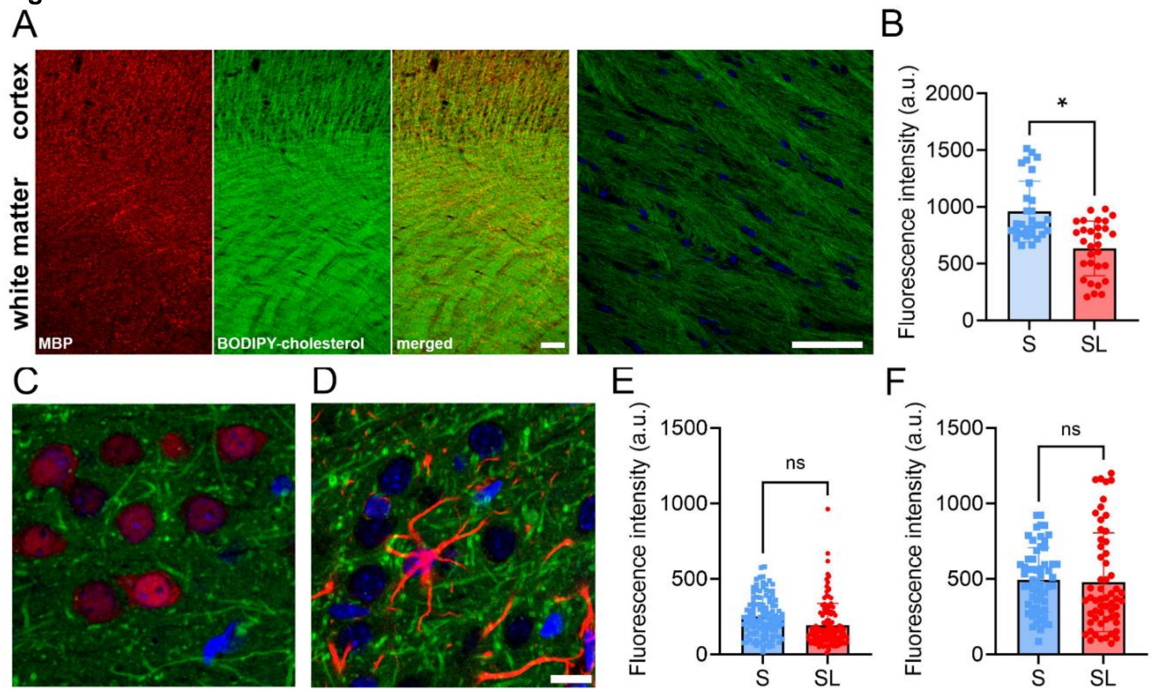

BODIPY-cholesterol levels in subcortical white matter, neurons, and astrocytes of S and SL rats. (A) From left to right. Representative images of BODIPY–cholesterol (green) and anti-myelin basic protein (MBP, red) staining in a rat. Scale bar: 50μm. Right. Representative image of BODIPY–cholesterol (green) and DAPI (blue) captured in the corpus callosum. Scale bar: 50μm. (B) Quantification of BODIPY-cholesterol fluorescence intensity in the corpus callosum of S (n = 30 fields, 5 rats) and SL (n = 30 fields, 5 rats) rats. *P<0.05. (C and D) Representative images of neurons (Neun+ cells, red in C) and astrocytes (GFAP+ cells in D, red) in association with BODIPY-cholesterol (green) and DAPI (blue). Scale bar: 10 μm. (E and F) BODIPY-cholesterol levels are estimated within the neuronal (E, S: n = 111 cells, 5 rats; SL: n = 125 cells, 5 rats) and astrocytic (F, S: n = 59 cells, 5 rats; SL: n = 58 cells, 5 rats) soma in S and SL rats.

Figure S12

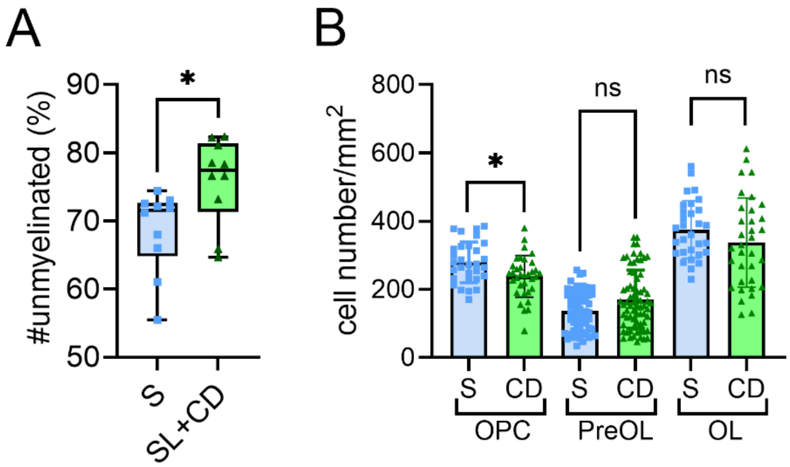

Effects of cyclodextrin on unmyelinated axons, OPCs, preOL, and OL densities in the corpus callosum. (A) Percentage of unmyelinated axons in S (n = 10 EM images, 3 rats) and SL+CD (n = 10 EM images, 3 rats). *P<0.05. (B) Quantification of OPC, preOL, and OL densities in S (OPC: n = 30, preOL: n = 60; OL: n = 30; 6 rats) and SL+CD (OPC: n = 34, preOL: n = 71; OL: n = 34; 7 rats) rats. *P<0.05. ns: not significant.

Dataset S1 (separate file). Up and downregulated probesets after SL.

Dataset S2 (separate file). Lipid expression data.

Dataset S3 (separate file). Source data and statistical details.

SI References

1. M. Bellesi, *et al.*, Effects of Sleep and Wake on Oligodendrocytes and Their Precursors. *J. Neurosci.* **33**, 14288–14300 (2013).
2. M. Bellesi, L. de Vivo, G. Tononi, C. Cirelli, Effects of sleep and wake on astrocytes: clues from molecular and ultrastructural studies. *BMC Biol.* **13**, 66 (2015).
3. S. Maret, U. Faraguna, A. B. Nelson, C. Cirelli, G. Tononi, Sleep and waking modulate spine turnover in the adolescent mouse cortex. *Nat. Neurosci.* **14**, 1418–1420 (2011).
4. J. W. Blanchard, *et al.*, APOE4 impairs myelination via cholesterol dysregulation in oligodendrocytes. *Nature* **611**, 769–779 (2022).
5. M. Nollet, Models of Depression: Unpredictable Chronic Mild Stress in Mice. *Curr. Protoc.* **1**, e208 (2021).
6. A. Sequeira-Cordero, A. Salas-Bastos, J. Fornaguera, J. C. Brenes, Behavioural characterisation of chronic unpredictable stress based on ethologically relevant paradigms in rats. *Sci. Rep.* **9**, 17403 (2019).
7. A. Leemans, B. Jeurissen, J. Sijbers, D. K. Jones, ExploreDTI: a graphical toolbox for processing, analyzing, and visualizing diffusion MR data in *Proc Intl Soc Mag Reson Med*, (2009), p. 3537.
8. Y. Assaf, P. J. Basser, Composite hindered and restricted model of diffusion (CHARMED) MR imaging of the human brain. *NeuroImage* **27**, 48–58 (2005).
9. S. M. Smith, *et al.*, Tract-based spatial statistics: Voxelwise analysis of multi-subject diffusion data. *NeuroImage* **31**, 1487–1505 (2006).
10. A. Klein, *et al.*, Evaluation of 14 nonlinear deformation algorithms applied to human brain MRI registration. *NeuroImage* **46**, 786–802 (2009).
11. P. Bankhead, *et al.*, QuPath: Open source software for digital pathology image analysis. *Sci. Rep.* **7**, 16878 (2017).
12. D. Bates, M. Maechler, B. Bolker, S. Walker, Fitting Linear Mixed-Effects Models Using lme4. *J. Stat. Softw.* **67**, 1–48 (2015).
13. A. Gramfort, *et al.*, MEG and EEG data analysis with MNE-Python. *Front. Neurosci.* **7** (2013).
14. E. Larson, *et al.*, MNE-Python. (2023). https://doi.org/10.5281/zenodo.7671973. Deposited 23 February 2023.
15. T. R. Derrick, B. T. Bates, J. S. Dufek, Evaluation of time-series data sets using the Pearson product-moment correlation coefficient. *Med. Sci. Sports Exerc.* **26**, 919 (1994).
16. T. Derrick, J. Thomas, Time Series Analysis: The Cross-Correlation Function. (2004).
17. S. Aydore, D. Pantazis, R. M. Leahy, A Note on the Phase Locking Value and its Properties. *NeuroImage* **74**, 231–244 (2013).
18. J.-P. Lachaux, E. Rodriguez, J. Martinerie, F. J. Varela, Measuring phase synchrony in brain signals. *Hum. Brain Mapp.* **8**, 194–208 (1999).
19. D. W. Huang, B. T. Sherman, R. A. Lempicki, Systematic and integrative analysis of large gene lists using DAVID bioinformatics resources. *Nat. Protoc.* **4**, 44–57 (2009).
20. J. N. Larocca, W. T. Norton, Isolation of myelin. *Curr. Protoc. Cell Biol.* **Chapter 3**, Unit3.25 (2007).
21. V. Matyash, G. Liebisch, T. V. Kurzchalia, A. Shevchenko, D. Schwudke, Lipid extraction by methyl-tert-butyl ether for high-throughput lipidomics. *J. Lipid Res.* **49**, 1137–1146 (2008).

22. A. Cutignano, *et al.*, Monitoring changes of lipid composition in durum wheat during grain development. *J. Cereal Sci.* **97**, 103131 (2021).
23. E. Manzo, M. L. Ciavatta, D. Pagano, A. Fontana, An efficient and versatile chemical synthesis of bioactive glyco-glycerolipids. *Tetrahedron Lett.* **53**, 879–881 (2012).
24. E. G. Bligh, W. J. Dyer, A rapid method of total lipid extraction and purification. *Can. J. Biochem. Physiol.* **37**, 911–917 (1959).
25. B. R. Lentz, Membrane "fluidity" as detected by diphenylhexatriene probes. *Chem. Phys. Lipids* **50**, 171–190 (1989).
26. L. A. Bagatolli, "LAURDAN Fluorescence Properties in Membranes: A Journey from the Fluorometer to the Microscope" in *Fluorescent Methods to Study Biological Membranes*, Springer Series on Fluorescence., Y. Mély, G. Duportail, Eds. (Springer, 2013), pp. 3–35.
27. M. Leger, *et al.*, Object recognition test in mice. *Nat. Protoc.* **8**, 2531–2537 (2013).
